# Research on the optimization of delivery routes for vehicles with drones under no-fly zone restrictions

**DOI:** 10.1371/journal.pone.0335614

**Published:** 2025-10-29

**Authors:** Yingbo Mao, Guiqiong Jia

**Affiliations:** School of management, Harbin University of Commerce, Harbin, China; Beijing Institute of Technology, CHINA

## Abstract

With the rapid development of e-commerce, logistics and distribution systems face the dual pressures of efficiency improvement and cost control. Unmanned Aerial Vehicle (UAV) delivery, featuring flexibility, high efficiency, and low carbon emissions, has become an effective means to solve the “last-mile” problem. However, the widespread no-fly zones in urban environments (e.g., airports, government agencies, and high-voltage power lines) severely limit the application scope of UAVs and increase the complexity of path planning. Against this backdrop, the vehicle-assisted UAV collaborative delivery model has emerged: through the division of labor and collaboration between ground vehicles and UAVs, it not only expands the service radius of UAVs but also overcomes the constraints of no-fly zones, achieving dual improvements in delivery efficiency and service quality.This study focuses on the optimization of vehicle-assisted UAV delivery paths under no-fly zone constraints, aiming to construct a multi-objective optimization model that balances delivery costs, carbon emissions, and customer satisfaction, and to design an efficient solution algorithm for providing scientific decision support to logistics enterprises. First, the paper systematically sorts out the classification and definition of no-fly zones as well as their impact mechanisms on UAV path planning, and elaborates on the theoretical basis of vehicle-UAV collaborative delivery, including the constituent elements of the problem, methods for quantifying customer satisfaction, and the application framework of heuristic algorithms. On this basis, a mixed-integer programming model is built with the objectives of minimizing total cost, minimizing carbon emissions, and maximizing customer satisfaction. Given that this model falls into the category of NP-hard problems, we have designed a four-stage heuristic solution. First, an improved K-means algorithm (IKM) is used to cluster customer points under the constraint of the UAV’s maximum flight radius, so as to determine vehicle parking points. Second, a multi-objective genetic algorithm is applied to plan UAV delivery routes for customers in open areas. Next, the multi-objective genetic algorithm is continued to design initial routes for vehicles between parking points. Finally, the multi-objective genetic algorithm is utilized again to plan delivery routes for customers in no-fly zones, ultimately forming a complete collaborative “vehicle-UAV” delivery scheme.To verify the effectiveness of the model and algorithm, simulation experiments are conducted using two sets of cases: 30 customer points in a local area of Harbin and the large-scale R201 case from the Solomon dataset. The results show that compared with traditional vehicle-only or UAV-only delivery models, the vehicle-UAV collaborative delivery model exhibits significant advantages in total cost, carbon emissions, and customer satisfaction; the model maintains good robustness in stability tests under different no-fly zone settings; and parameter sensitivity analysis further reveals the impact of key parameters (e.g., UAV load capacity, endurance, and vehicle load capacity) on delivery performance, providing practical references for logistics enterprises in equipment selection and operation strategy formulation.

## 1. Introduction

The explosive growth of e – commerce and consumers’ urgent demand for instant delivery have made the “last - mile” problem of logistics delivery a focus of attention in academia and industry. The traditional vehicle delivery model has difficulty meeting the efficient and low – carbon logistics requirements due to defects such as traffic congestion, high carbon emissions, and the rigidity of fixed routes. Drones (Unmanned Aerial Vehicle, UAV), with their flexible take – off and landing, low – altitude obstacle avoidance, and rapid response capabilities, are regarded as a key technology to break through the delivery bottleneck [1,2]. However, drones face multiple challenges in practical applications, such as endurance limitations [3], load capacity constraints [4], and airspace control (e.g., no – fly zones) [5]. The single – drone delivery model is difficult to cover complex scenarios. Therefore, the collaborative delivery of vehicles and drones (Truck – Drone Collaborative Delivery, TDCD) has become a research hotspot. Murray [6] first proposed the vehicle routing problem with drones.It achieves a balance among cost, timeliness, and coverage through resource complementarity [7,8] and has gradually become an important direction for the optimization of modern logistics systems.

In terms of theoretical model construction, early research focused on the basic framework of drone route optimization. Dorling [1] first proposed the multi – trip drone route problem (VRP), optimizing energy consumption and delivery time through mixed – integer programming and simulated annealing algorithms, but did not consider the hard constraints of no – fly zones on the route. hiang [2] constructed a green routing model from the perspective of sustainability, proving that drones can significantly reduce carbon emissions. However, its static order assumption is difficult to adapt to the dynamic logistics environment. Dukkanci [9] systematically sorted out the four collaborative modes of TDCD (parallel, hybrid, truck – assisted drone, and drone – assisted truck), pointing out that the dynamic environment and multi – type vehicle collaboration are the future research directions. However, most existing research focuses on fixed networks and idealized constraints, and there are still obvious gaps in the joint optimization of no – fly zones and real – time order changes [10,11].

In terms of algorithm design and solution, scholars have attempted to combine traditional combinatorial optimization methods with emerging intelligent algorithms. Toy [12] proposed a collaborative scheduling model with trucks as mobile take – off and landing platforms, minimizing the total delivery time through mixed – integer programming, but did not involve route clustering and dynamic adjustment. Baldisseri [13] developed an ant colony optimization (ACO) algorithm to solve the drone-truck pairing problem, achieving a 30% cost savings. However, its clustering process depends on fixed customer grouping and lacks self – adaptability. Kischst [14] proposed a heterogeneous multi – drone collaborative framework, optimizing delivery efficiency through fuzzy C – means clustering and variable neighborhood descent algorithms, but did not consider the route avoidance requirements of no – fly zones.Weng [15] proposed a new two-stage hybrid heuristic algorithm, where the first stage uses an improved K-means clustering algorithm to cluster temporary substations; the second stage optimizes the routes of trucks and drones by combining Tabu Search (TS), Enhanced Genetic Algorithm (GA), and Simulated Annealing (SA).

In addition, dynamic order processing has become a recent research difficulty. Huang [16] used the deep Q – learning method to achieve real – time scheduling, but its model complexity is high and difficult to apply to large – scale scenarios. Marine [17] proposed a variable neighborhood search (VNS) to improve the periodic flight of drones, but the no – fly processing is limited to single – point avoidance and has not been extended to regional – level constraints.

As the core element of airspace control, no – fly zones directly determine the feasibility of drone routes. Eskan [18] reviewed the facility location problem of drone – riding vehicles, emphasizing that the intersection point planning needs to take airspace restrictions into account, but did not propose a dynamic collaborative mechanism. Benarbia [19] proposed the optimization of charging station deployment, balancing endurance and cost through a MINLP model, but its route planning is not linked with no – fly zones. Chang [20] studied the collaborative delivery of drones and public transportation, constructing a charging station deployment model, but did not solve the dynamic avoidance problem of no – fly zones. Liu [21] constructed a mixed-integer linear programming (MILP) model that considers no-fly zones and the joint service path optimization problem for trucks and drones that handle both pickup and delivery.

Wang [22] reviewed 95 papers on mixed truck – drone delivery and pointed out that only 12% of the research involved airspace restrictions, and most of them adopted static avoidance strategies. Existing models such as Huang [23] genetic algorithm only deal with fixed no – fly zones and lack dynamic collaboration with vehicle routes.However, In recent research directions such as reinforcement learning, graph neural networks, and multi-agent cooperation, Chen [24] personalized longitudinal motion planning based on reinforcement learning and imitation learning methods, considering multiple performance indicators as well as the driver’s acceptance of the vehicle’s driving style. Chen [25] proposed an integrated eco-driving framework for fuel cell hybrid electric vehicles in a multi-lane highway scenario, synchronously optimizing the delivery route through a unified continuous control variable.

As a key indicator of logistics service, most existing models focus on time – window penalties [26], while ignoring the exponential impact of delivery timeliness on customers’ psychology. Crisan [27] proposed a multi – objective optimization model that combines carbon emissions and time cost, but its satisfaction function does not quantify customers’ psychological expectations. Boysen [28] pointed out through empirical analysis that customers’ acceptance of drones is closely related to delivery speed and privacy risks, and a more practical satisfaction model needs to be constructed. Li [29] compared the environmental impacts of drones and motorcycles, proving that low – carbon delivery can indirectly improve customer satisfaction, but did not establish a direct correlation function.

In response to the above problems, this paper proposes a route optimization model for vehicle – drone joint delivery under no – fly zone constraints, with the following innovations:

(1) It constructs a multi-objective optimization model for vehicle-UAV collaborative delivery under no-fly zone constraints, overcoming the limitation of ignoring airspace restrictions in traditional path optimization studies.
(2) It proposes a phased solution strategy that integrates an improved clustering algorithm with multiple meta-heuristic algorithms, effectively enhancing the solution efficiency and quality for large-scale problems under complex constraints.
(3) It verifies the applicability and stability of the model and algorithm in real-world scenarios through empirical analysis, providing theoretical support and practical guidance for logistics enterprises to promote intelligent and green delivery.

The structure of this article is as follows: Chapter 2 constructs a vehicle delivery route optimization model with drones under the constraint of no-fly zones; Chapter 3 details the design and implementation process of the multi-stage algorithm; Chapter 4 verifies the effectiveness of the model through small-scale and large-scale examples, and analyzes the sensitivity of parameters and the model performance in different scenarios; Chapter 5 summarizes the research conclusions and presents future research directions. Based on existing research, this study provides a theoretical foundation for the vehicle delivery route planning problem with drones by considering the constraint of no-fly zones, as well as a decision support tool for logistics companies that offers low costs and high satisfaction.

## 2. Vehicle-assisted drone delivery route optimization considering no-fly conditions

### 2.1 Problem description

This study plans vehicle-assisted UAV collaborative delivery routes under the constraint that there are UAV no-fly zones in the delivery area. The problem can be described as follows: A logistics enterprise establishes a mixed fleet consisting of vehicles and UAVs to perform delivery tasks. Before delivery, vehicle parking points are first determined based on the results of customer point clustering. Vehicles, carrying UAVs and goods, depart from the distribution center and pass through vehicle parking points in sequence. At each parking point, UAVs take off from the vehicle to deliver goods to customer points in the open area belonging to that parking point. After all UAVs have completed deliveries to customers in the open area and returned to the vehicle, the vehicle then delivers goods to customer points located within no-fly zones. Subsequently, the vehicle, carrying the UAVs, proceeds to the next parking point to continue the delivery task until all customer points have been served, and finally returns to the distribution center.

In the delivery area of the logistics enterprise, there are restrictions imposed by no-fly zones: customers within no-fly zones cannot be served by UAVs and must be served by vehicles. Therefore, this study establishes a route planning model for vehicle-assisted UAV delivery and formulates a scientific and reasonable path optimization scheme to achieve the goals of minimizing logistics operation costs, minimizing carbon emissions, and maximizing customer satisfaction.

### 2.2 Model assumptions

Assumption 1: Each customer belongs to only one clustering point (cluster center).
Assumption 2: The cluster center is the vehicle stopping point, and the cluster center does not coincide with the customer point.
Assumption 3: There is only one distribution center.
Assumption 4: Each vehicle carries m on-board UAVs.
Assumption 5: The flight routes of UAVs for delivery are Manhattan routes.
Assumption 6: Under the constraints of drone carrying capacity and endurance, the drone can continuously deliver to multiple customer points.
Assumption 7: The flight speed of a UAV during delivery is inversely proportional to its load weight.
Assumption 8: Customer points within no-fly zones are served by vehicles, while customer points in open areas are served by UAVs.
Assumption 9: Customers in open areas are referred to as Type 1 customers, and customer points within no-fly zones are referred to as Type 2 customer points. If a vehicle parking point has Type 2 customer points, the vehicle must wait for the UAVs to finish delivering to Type 1 customers and return to the vehicle before delivering to Type 2 customers; thereafter, the vehicle proceeds to the next parking point.
Assumption 10: For the convenience of calculation, this paper uses the Manhattan distance for calculating the UAV flight distance between two points. However, in an environment with complex no-fly zones, the length of the UAV’s actual detour path may be much longer than the Manhattan distance.

### 2.3 Model parameters and variables

The model parameters constructed in this paper are mainly divided into three categories: constants, sets, and variables. The specific symbols and their meanings are shown in Table 1.

**Table 1:** Meanings of model symbols.

	Symbol	Meaning
Constant	C=0(center)	Denotes the number of the distribution center
	FC	Denotes the fixed cost per vehicle
	$c_{\text{0}}$	Denotes the cost per unit fuel consumption
	$c_{\text{1}}$	Denotes the cost per unit flight distance of the UAV
	$c_{\text{2}}$	Denotes the time penalty cost coefficient for early arrival
	$c_{\text{3}}$	Denotes the time penalty cost coefficient for late arrival
	$q_{i}$	Denotes the demand volume of the customer point i
	$Q_{s}$	Denotes the total demand volume of all customers at parking point s
	$W_{k}$	Denotes the maximum load capacity of the vehicle
	$V_{t}$	Denotes the driving speed of the vehicle
	$W_{d}$	Denotes the driving speed of the vehicle
	${[V}_{d}^{l},V_{d}^{r}]$	Denotes the flight speed of the UAV under full-load and no-load conditions
	$L_{k}$	Denotes the maximum driving distance of the vehicle
	θ	Denotes the fuel consumption coefficient per unit driving distance of the vehicle
	$L_{d}$	Denotes the maximum flight distance of the UAV
	$L_{ij}$	Denotes the driving distance of the vehicle from node i to node j
	$D_{ij}$	Denotes the 3D flight distance of the UAV from node i to node j
	$[tl_{i},{tr}_{i}]$	Denotes the required delivery time window of the customer point
	$[t_{i}^{\prime },T_{i}]$	Denotes the arrival time and departure time at the node i
	$T_{s}$	Fixed value, denotes the service time at the customer point
Sets	S={1,2,...,s}	Set of cluster centers (vehicle parking points)
	$N=\{N_{\text{1}},N_{\text{2}},...,N_{s}\}$ $N_{s}=\phi_{s}\cup \psi_{s}$	Set of customer points under cluster center s
	$\phi =\{\phi_{\text{1}},\phi_{\text{2}},...,\phi_{s}\}$	Set of Type 1 customer points (in open areas) at all parking points
	$\psi =\{\psi_{\text{1}},\psi_{\text{2}},...,\psi_{s}\}$	Set of Type 2 customer points (in no-fly zones) at all parking points
	M={1,2,…,m}	Set of numbers of all customer points
	K={1,2,…,k}	Set of numbers of all vehicles
	${VD}_{d}^{k}=\{{VD}_{\text{1}}^{k},{VD}_{\text{2}}^{k},...,{VD}_{d}^{k}\}$	Set of UAVs carried by vehicle k
	H=C∪M∪S	Set of all nodes
	G=C∪S	Set of the distribution center and all parking points
	$R_{r}=\{r|r=\text{1},\text{2},\ldots r\}$	Set of no-fly zones
Variables	$x_{ij}^{k}=\{\text{0},\text{1}\}$	Binary variable; equals 1 if vehicle k travels from node i to node j, otherwise 0
	$y_{ij}=\{\text{0},\text{1}\}$	Binary variable; equals 1 if delivery from node i to node j is performed by a UAV, otherwise 0
	$R_{m}=\{\text{0},\text{1}|m\in M\}$	Binary variable; equals 1 if customer point m is a Type 1 customer point, otherwise 0
	$z_{s}^{k}=\{\text{0},\text{1}\}$	Binary variable; equals 1 if parking point s is served by vehicle k,otherwise 0

### 2.4 Construction of UAV flight speed function model

In practice, it is common for UAVs to have speed differences due to varying weights of delivered goods. Therefore, this paper assumes that the flight speed of a UAV under full load (i.e., when the UAV is at its maximum load capacity) is $V_{d}^{l}$, and its flight speed under no load (i.e., when the UAV has zero load) is $V_{d}^{r}$; the speed of both vehicles and UAVs during delivery is inversely proportional to their load weight (refer to Assumption 7). Thus, the flight speed of a UAV when delivering goods with weight w can be expressed as follows:


$${\text{V}}_{\text{d}}={\text{V}}_{\text{d}}^{\text{r}}-\frac{{\text{V}}_{\text{d}}^{\text{r}}-{\text{V}}_{\text{d}}^{\text{l}}}{{\text{W}}_{\text{d}}}*\text{w}$$ (1)

### 2.5 UAV route planning model considering no-fly zones

**Fig 1:**
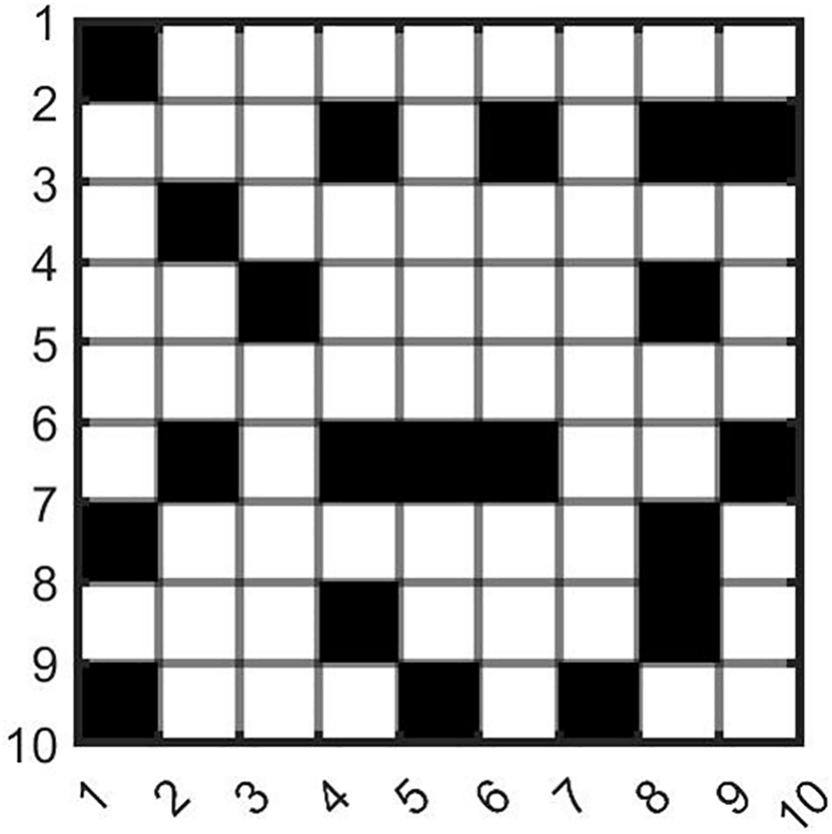
Schematic diagram of no-fly zones.

According to Assumption 5, UAV delivery routes follow the Manhattan distance principle. For the convenience of planning, this paper adopts the grid method to divide the delivery area into 2D grids and 3D grids for analysis. Given that there are no-fly zones in the delivery area (in this paper, no-fly zones only affect UAV delivery and have no impact on vehicle delivery), values are assigned to grids to define whether they belong to no-fly zones. As shown in Fig 1, white grids represent open areas, while black grids represent no-fly zones.

The grid method is a spatial discretization representation method, often used in fields such as path planning and environment modeling. It divides continuous space into regular grid units, and each grid can be assigned different attributes. Through the operation and analysis of grids, it realizes the modeling of spatial environments and related tasks, converting complex spatial problems into computable problems based on discrete grids. For example, in UAV delivery scenarios, dividing the delivery area into grids facilitates the planning of obstacle-avoiding flight paths. As shown in equation (2), black grids are defined as no-fly areas and assigned a value of 1, while white grids are defined as flyable areas and assigned a value of 0.

Let the 2D grid matrix be $G_{\text{2}D}$ with a size of m × n, and the grid coordinates be represented by (x,y). Then, the state of a grid can be expressed as equation (2):


$${\text{G}}_{\text{2D}}[\text{i}][\text{j}]=\left\{ \begin{array}{c} @l\text{1},\text{black}\;\text{grids}\;\text{represent}\;\text{no}-\text{fly}\;\text{zones}\\ \text{0},\text{white}\;\text{grids}\;\text{represent}\;\text{open}\;\text{areas} \end{array}\right.\;$$ (2)

To calculate the 3D flight distance of the UAV, first, this paper defines the Manhattan distance of the UAV between the start point and end point in the 2D grid is shown in equation (3):


$${\text{d}}_{\text{Manhattan}-\text{2D}}=\Gamma (\text{S},\text{E})=\Gamma (({\text{x}}_{\text{s}},{\text{y}}_{\text{s}}),({\text{x}}_{\text{e}},{\text{y}}_{\text{e}}))$$ (3)

Here, $\Gamma ((x_{s},y_{s}),(x_{e},y_{e}))$ refers to the sum of the total horizontal movement distance and total vertical movement distance of the UAV from the start point to the end point. This is because obstacle avoidance is required for UAV flight due to the existence of no-fly zones, so the optimal path is searched from four directions. However, this paper does not discuss how to find obstacle-avoiding paths, but only focuses on calculating the distance of such paths.

**Fig 2:**
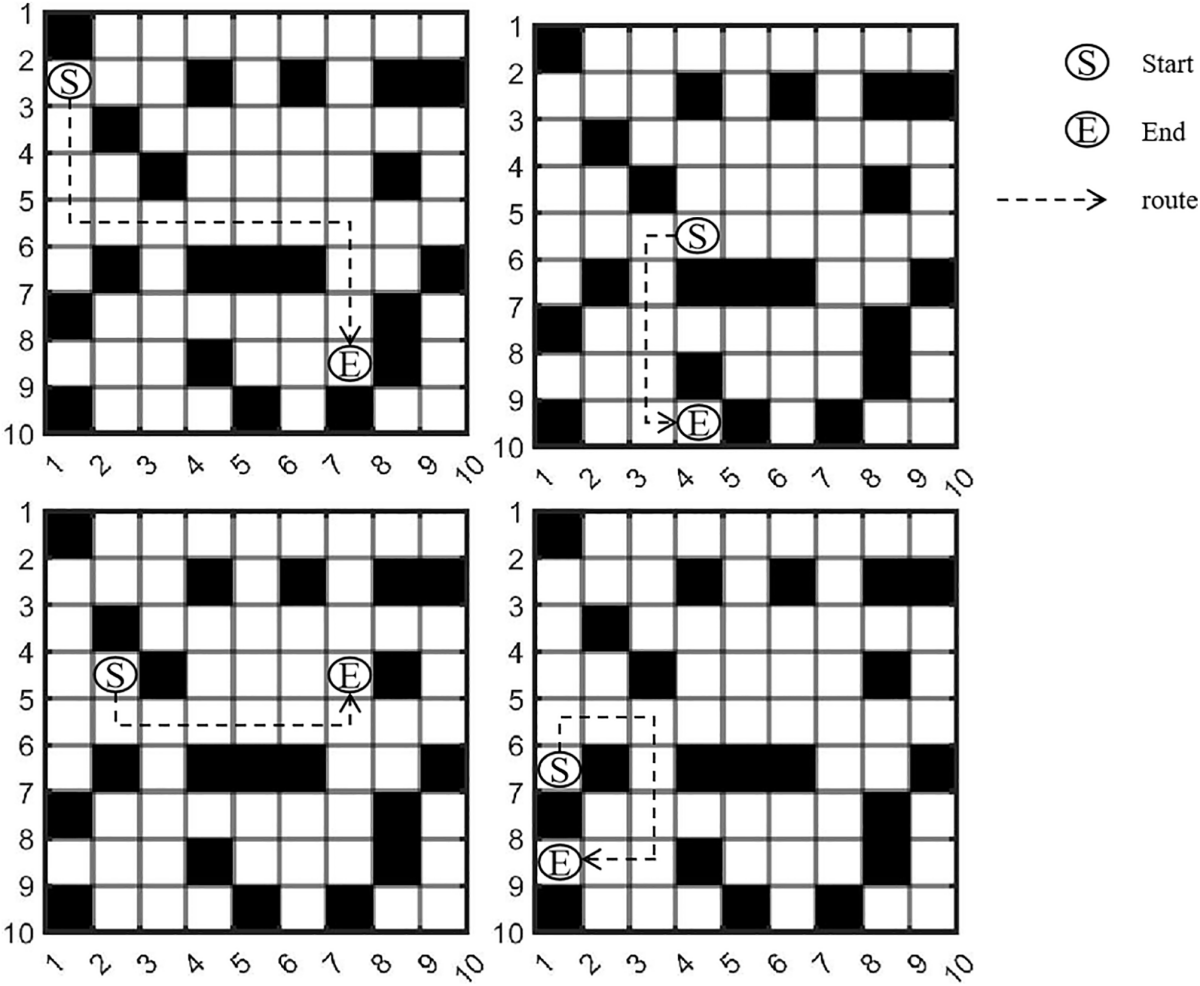
Schematic diagram of obstacle-avoiding paths.

**Fig 3:**
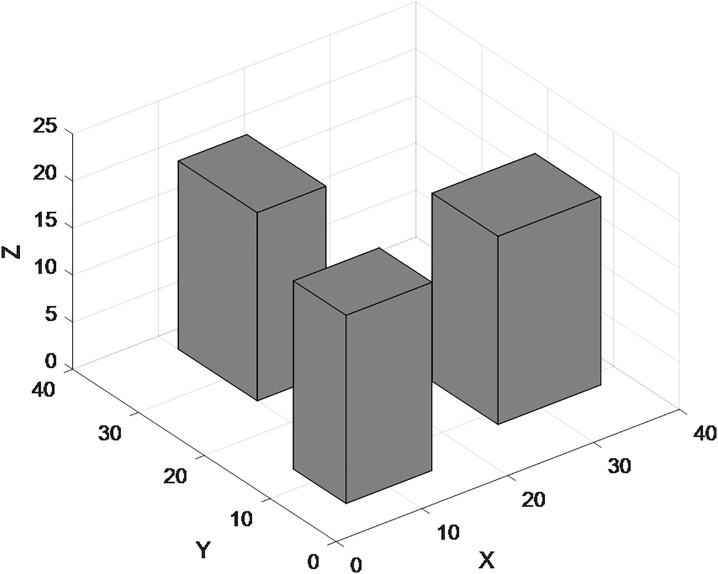
Schematic diagram of 3D no-fly zones.

The Fig 2 clearly show that the Manhattan distance from the start point to the end point is actually the sum of the total horizontal movement distance and total vertical movement distance between the two points. However, in a 3D environment (as shown in Fig 3), there is an additional “height” (Z-axis) compared to the 2D environment. UAVs need to travel along “vertical lines” or “diagonal lines” during takeoff and landing. Therefore, the flight path planning in 3D grids can be divided into two steps: horizontal obstacle-avoiding route in the air + takeoff and landing routes.

**Fig 4:**
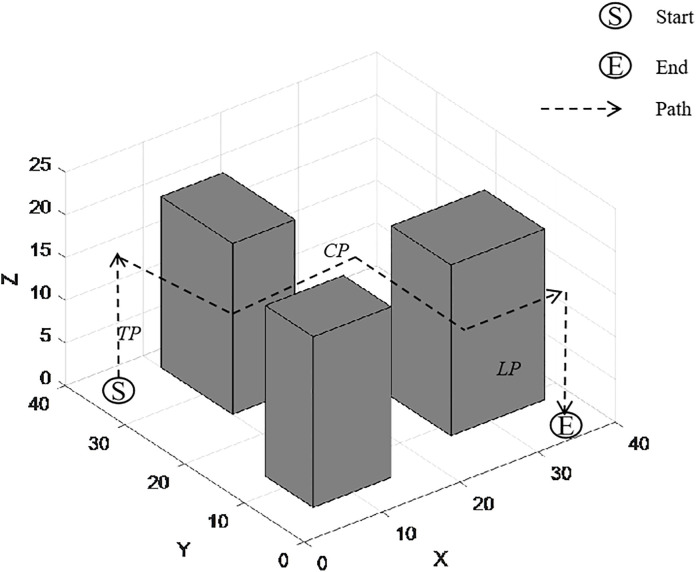
Flight path in three-dimensional space.


Thus, the 3D flight (as shown in Fig 4) can be split into three phases for analysis: the Takeoff Phase (TP), the Cruise Phase (CP), and the Landing Phase (LP):

Takeoff Phase (TP): From the ground to the cruise altitude, following a “vertical” takeoff path.
Cruise Phase (CP): From point A to point B, following an obstacle-avoiding path in the 2D grid (horizontal movement with constant altitude).
Landing Phase (LP): From the cruise altitude to the ground at the target point, following a “vertical” landing path.

For the Takeoff Phase and Landing Phase, it is assumed that the UAV takes off and lands vertically (i.e., the altitude changes from 0 to H). The physical flight distances of the Takeoff Phase and Landing Phase are the same. The distance of the Takeoff Phase is shown in equation (4):


$${\text{D}}_{\text{Takeoff}}=\int_{\text{0}}^{{\text{T}}_{\text{takeoff}}}\sqrt{{(\frac{\text{dx}}{\text{dt}})}^{\text{2}}+{(\frac{\text{dy}}{\text{dt}})}^{\text{2}}+{(\frac{\text{dz}}{\text{dt}})}^{\text{2}}}\text{dt}$$ (4)

By substituting the horizontal movement speed $\frac{dx}{dt}=\text{0}$, $\frac{dy}{dt}=\text{0}$, vertical speed $\frac{dz}{dt}=\text{a}t$, and T (representing takeoff time), the equation (5) can be obtained:

$${\text{D}}_{\text{Takeoff}}=\int_{\text{0}}^{\text{T}}\sqrt{\text{0}+\text{0}+{\left(\text{at}\right)}^{\text{2}}}\text{dt}=\int_{\text{0}}^{\text{T}}\text{atdt}=\frac{\text{1}}{\text{2}}\text{a}{\text{T}}^{\text{2}}$$ (5)

Let *H*=$\frac{\text{1}}{\text{2}}\text{a}T^{\text{2}}$, Therefore, the total 3D distance of the UAV between two points is equal to the sum of the Takeoff Phase distance, Cruise Phase distance, and Landing Phase distance, which is shown in equation (6):


$${\text{D}}_{\text{3D}-\text{Physical}\;}={\text{D}}_{\text{takeoff}}+{\text{D}}_{\text{cruise}}+{\text{D}}_{\text{landing}}=\text{2H}+(|{\text{x}}_{\text{e}}-{\text{x}}_{\text{s}}\left|+\right|{\text{y}}_{\text{e}}-{\text{y}}_{\text{s}}\left|\right)$$ (6)

### 2.6 Cost function construction

In the model constructed in this paper, the cost consists of three components: the cost incurred by vehicle delivery, the cost of drone delivery, and the time penalty cost caused by early or late delivery. This is shown in Equation (7) below:.


$$\text{F}=F_{\text{1}}+F_{\text{2}}+F_{\text{3}}$$ (7)

Where $F_{\text{1}}$ represents the delivery cost generated by vehicle delivery, $F_{\text{2}}$ represents the delivery cost generated by drone delivery, and $F_{\text{3}}$ represents the time penalty cost for early or late arrival at customer points.

(1) The delivery cost incurred by vehicles mainly consists of two parts, as shown in Equation (8):.

$$F_{\text{1}}=f_{\text{1}}+f_{\text{2}}$$ (8)

where $f_{\text{1}}$ represents the fixed cost of the vehicle, which usually refers to the basic, normal and inevitable costs related to vehicle use. These costs do not fluctuate significantly with the vehicle’s traveling mileage or usage time. Therefore, the fixed cost of a single vehicle delivery per unit is set as a fixed value FC.


$$f_{\text{1}}=F\text{C}\sum_{k\in K}\sum_{j\in U}x_{Cj}^{k}$$ (9)

In Equation (9), C represents the distribution center, G=C∪S represents the set of vehicle parking points and customer points, FC represents the fixed cost per vehicle, and $\sum_{k\in K}\sum_{j\in G}x_{Cj}^{k}$ represents the number of vehicles dispatched from the distribution center to the set of vehicle parking points and customer points for the first stop..

$f_{\text{2}}$ represents the transportation cost incurred by vehicles, which mainly refers to the cost generated by fuel consumption. Its calculation method is the product of the vehicle’s driving distance, the fuel consumption coefficient per unit distance, and the cost per unit fuel consumption:.


$$f_{\text{2}}=c_{\text{0}}\theta \sum_{k\in K}\sum_{j\in G\cup \phi }\sum_{i\in G\cup \phi }{d_{ij}x}_{ij}^{k}$$ (10)

In Equation (10), $c_{\text{0}}$ Represents the cost per unit fuel consumption, θ Represents the vehicle fuel consumption coefficient per unit distance, H=C∪M∪S represents the set of all nodes,and $\sum_{k\in K}\sum_{j\in G\cup \phi }\sum_{i\in G\cup \phi }{d_{ij}x}_{ij}^{k}$ represents the total driving distance of all vehicles.

(2) The costs generated from drone delivery are primarily the transportation costs of the drone. The unit flight distance cost of the drone is understood as the electricity consumption during flight, various acquisition configurations, and other expenses, with these costs averaged out over the flight distance. The calculation method for drone transportation costs is the product of the drone’s flight distance and the unit flight distance cost of the drone.

$$F_{\text{2}}=c_{\text{1}}\sum_{d\in D}\sum_{k\in K}\sum_{j\in G}\sum_{i\in G}{VD}_{d}^{k}D_{ij}{\text{y}}_{\text{ij}}$$ (11)

In Equation (11), ${VD}_{d}^{k}$ represents the set of on-board UAVs carried by the vehicle, and $\sum_{d\in D}\sum_{\text{k}\in \text{K}}\sum_{j\in \text{G}}\sum_{i\in G}{VD}_{d}^{k}{\text{D}}_{\text{ij}}y_{ij}$ represents the total flight distance of all UAVs.

(3) The time penalty cost for customer points in this study refers to the cost incurred when goods are delivered earlier than the earliest required time or later than the latest required time by vehicles or UAVs. In such cases, the time delay cost for the customer point is the product of the unit time penalty cost coefficient and the delay duration.

**Fig 5:**
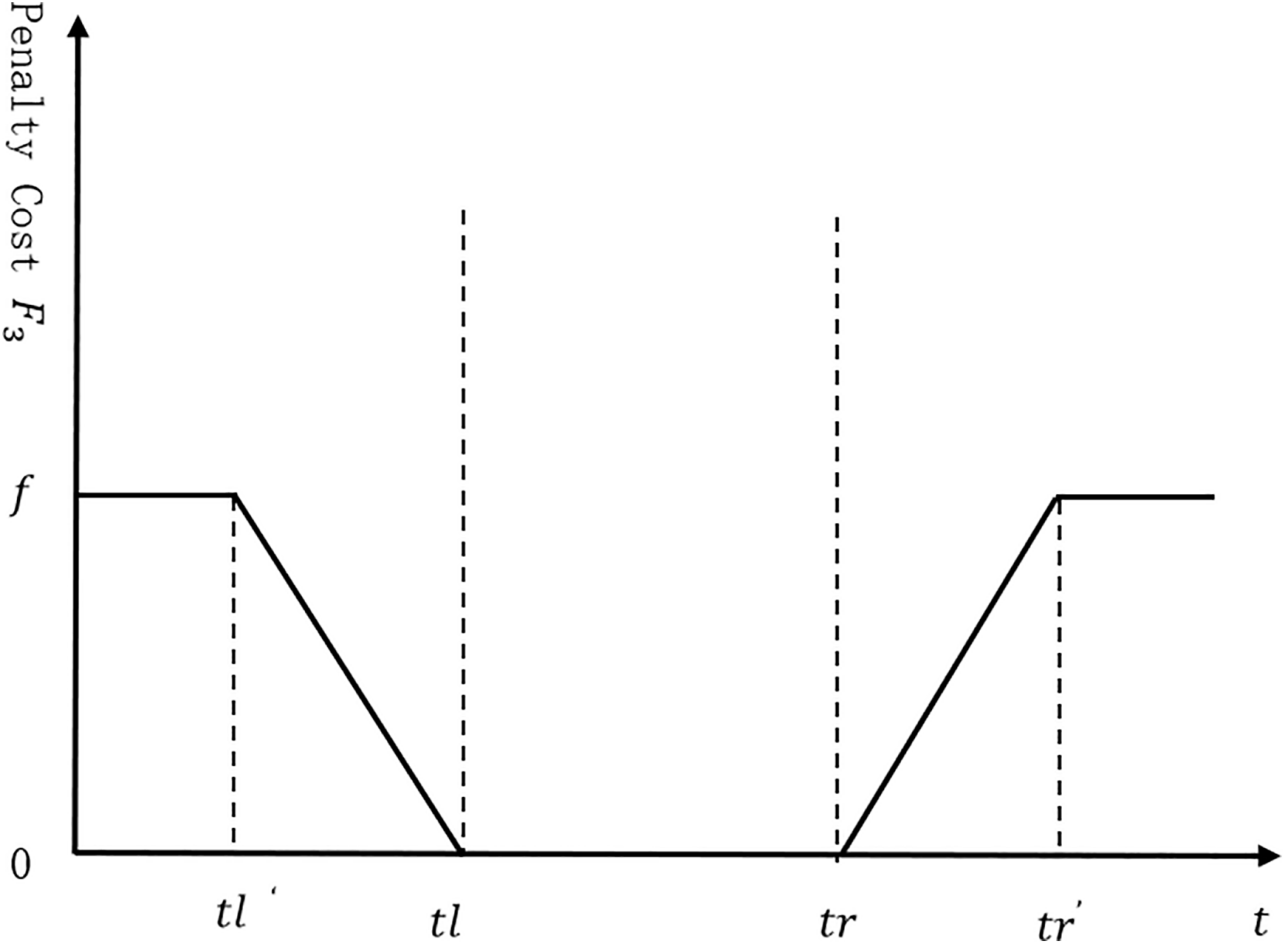
Relationship between delivery time and penalty cost.


As shown in Fig 5, the calculation formula for the time penalty cost of customer point i is given by Equation (12) as follows:

$${\text{w}}_{\text{i}}=\left\{ \begin{array}{c} @c\text{f},{\;\;\;\text{t}}_{\text{i}}^{\prime }\leq {\text{tl}}_{\text{i}}^{\prime }\\ {\text{c}}_{\text{2}}∙({\text{tl}}_{\text{i}}-{\text{t}}_{\text{i}}^{\prime }),\;\;\;{\text{tl}}_{\text{i}}^{\prime }\leq {\text{t}}_{\text{i}}^{\prime }\leq \text{tl}\\ \text{0},\;\;\;\text{tl}\leq {\text{t}}_{\text{i}}^{\prime }\leq \text{tr}\\ {\text{c}}_{\text{3}}∙({\text{t}}_{\text{i}}^{\prime }-\text{t}{\text{r}}_{\text{i}}),\;\;\;\text{tr}\leq {\text{t}}_{\text{i}}^{\prime }\leq {\text{tr}}_{\text{i}}^{\prime }\\ \text{f},{\;\;\;\text{t}}_{\text{i}}^{\prime }>{\text{tr}}_{\text{i}}^{\prime } \end{array}\right.\;$$ (12)

In Equation (12), $[{\text{tl}}_{\text{i}}^{\prime },{\text{tr}}_{\text{i}}^{\prime }]$ represents the earliest and latest acceptable delivery times for customer i; ${\text{tl}}_{\text{i}}-t_{i}^{\prime }$ represents the difference between the actual delivery time to the customer point and the earliest required time by the customer point; and $t_{i}^{\prime }-{tr}_{i}$ represents the difference between the actual delivery time to the customer point and the latest required time by the customer point.Then, the calculation formula for the total time penalty cost of all customer points is given by Equation (13) as follows:


$${\text{F}}_{\text{3}}=\sum_{\text{i}\in \text{M}}{\text{w}}_{\text{i}}$$ (13)

### 2.7 Construction of carbon emission function

Vehicle delivery consumes fuel, which in turn generates carbon emissions. However, drone delivery consumes electricity and does not produce carbon emissions. According to relevant literature, the ratio of unit fuel consumption to the resulting carbon emissions is approximately 2.3 [30].Therefore, in this paper, the calculation formula for carbon emissions is given in Equation (14) as follows:


$$\Omega =\frac{\theta }{\text{2}.\text{3}}\sum_{k\in K}\sum_{j\in G\cup \phi }\sum_{i\in G\cup \phi }{d_{ij}x}_{ij}^{k}$$ (14)

### 2.8 Construction of the customer satisfaction function model

Factors affecting customer satisfaction mainly include product quality, service quality, delivery experience, price factors, and the speed of delivery time. In the model of this paper, the focus is placed on the impact of delivery time on customer satisfaction.

**Fig 6:**
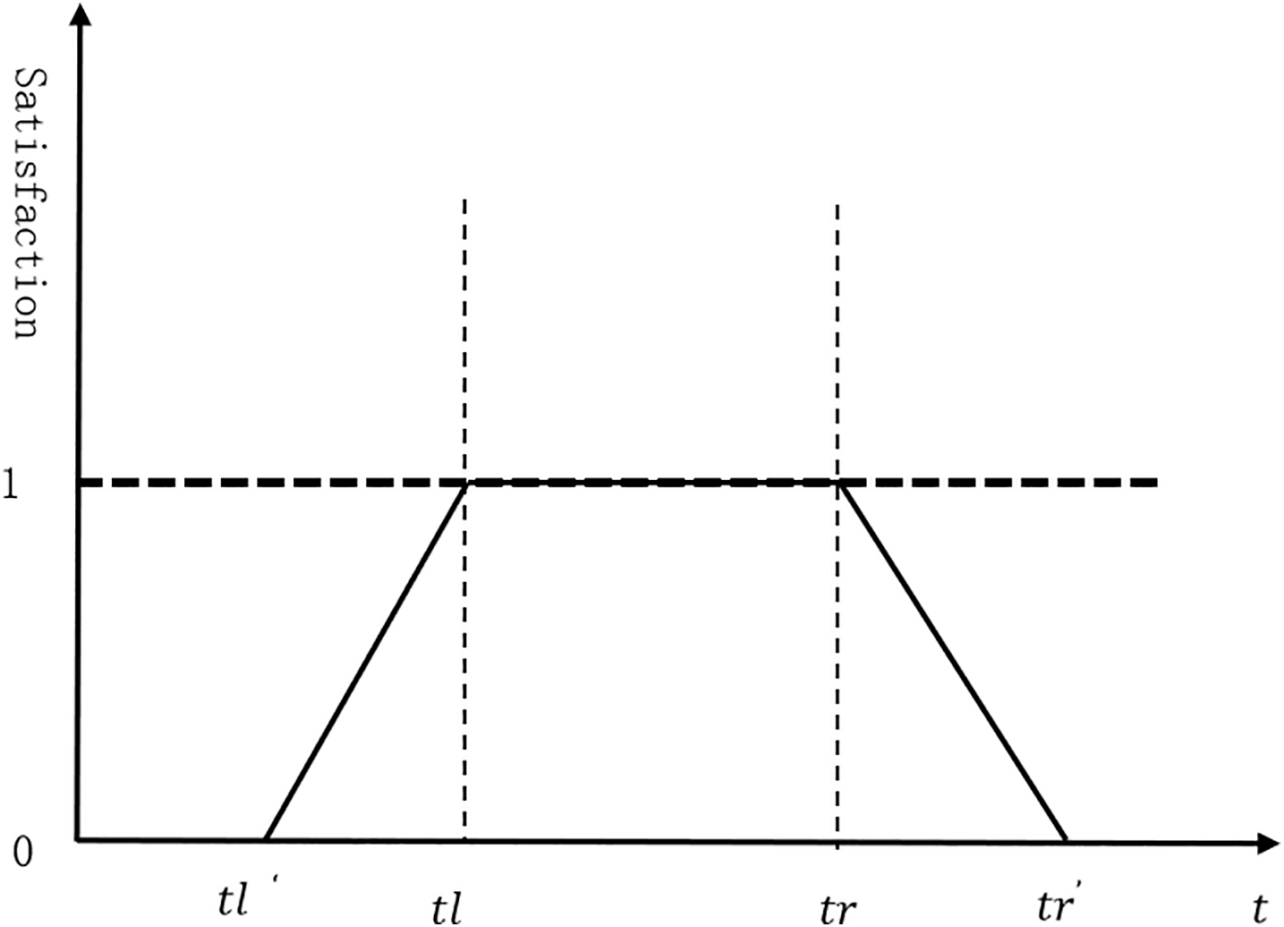
Relationship between delivery time and customer satisfaction.


As shown in Fig 6, the function describing the impact of delivery time on the satisfaction of customer point i is presented in Equation (15) below:

$${\text{P}}_{\text{i}}=\left\{ \begin{array}{c} @l\text{0},{\;\;\;\text{t}}_{\text{i}}^{\prime }\leq {\text{tl}}^{\prime }\\ \frac{{\text{t}}_{\text{i}}^{\prime }}{{\text{tl}}_{\text{i}}},\;\;\;{\text{tl}}^{\prime }\leq {\text{t}}_{\text{i}}^{\prime }\leq \text{tl}\\ \text{1},\;\;\;\text{tl}\leq {\text{t}}_{\text{i}}^{\prime }\leq \text{tr}\\ \frac{{\text{t}}_{\text{i}}^{\prime }}{{\text{tr}}_{\text{i}}},\;\;\;\text{tr}\leq {\text{t}}_{\text{i}}^{\prime }\leq {\text{tr}}^{\prime }\\ \text{0},{\;\;\;\text{t}}_{\text{i}}^{\prime }>{\text{tr}}^{\prime } \end{array}\right.\;$$ (15)

As indicated in the above equation (15), when the goods are delivered to customer point i by vehicle or drone within the time window required by the customer, the satisfaction of customer point i is 1. If the delivery is earlier or later than the required time window, the satisfaction decreases linearly. When the delivery exceeds the maximum acceptable time window of the customer point, the satisfaction is 0. In this paper, the final goal is to calculate the average satisfaction of all customer points, and it is shown in Equation (16):


$$\text{P}=\frac{\text{1}}{\text{M}}\sum_{\text{i}\in \text{M}}{\text{P}}_{\text{i}}$$ (16)

### 2.9 Model establishment

Optimization Objectives:


min(F)=F (17)

$$\begin{array}{c} \text{min}\left(\text{Ω}\right)=\text{Ω} \end{array}$$ (18)

$$\text{max}\left(P\right)=\frac{\text{1}}{\text{M}}\sum_{i\in M}P_{i}$$ (19)

Constraints:


$$\sum_{k\in K}\sum_{\text{i}\in \text{H}}\left({\text{x}}_{\text{ij}}^{\text{k}}+{\text{y}}_{\text{ij}}\right)=\text{1},\text{j}\in \text{M}$$ (20)

$$N_{\text{1}}\cup N_{\text{2}}\cup \ldots \cup N_{s}=M$$ (21)

$$N_{i}\cap N_{j}=\emptyset ,i\neq j;i,j\in s$$ (22)

$$\sum_{\text{s}\in \text{S}}{\text{Q}}_{\text{s}}{\text{Z}}_{\text{s}}^{\text{k}}\leq W_{k},k\in K$$ (23)

$$\sum_{j\in \text{M}}\sum_{i\in (M\cup S)}y_{ij}q_{j}\leq W_{d},d\in {VD}_{d}^{k}$$ (24)

$$\sum_{j\in H}\sum_{i\in \text{H}}x_{ij}^{k}d_{ij}\leq L_{k},k\in K$$ (25)

$$\sum_{j\in (M\cup S)}\sum_{i\in (M\cup S)}{\text{y}}_{\text{ij}}{\text{d}}_{\text{ij}}\leq L_{d},d\in {VD}_{d}^{k},\text{k}\in K$$ (26)

$$\sum_{i\in G}x_{Ci}^{k}-\sum_{j\in G}x_{jC}^{k}=\text{0},k\in K$$ (27)

$$(\sum_{i\in \phi_{s}}{\text{y}}_{\text{si}}-\sum_{j\in \phi_{s}}{\text{y}}_{\text{js}})z_{s}^{k}=\text{0},k\in K,s\in S$$ (28)

$$\sum_{i\in N}x_{si}^{k}=\sum_{j\in N}x_{js}^{k}=\text{1},k\in K,s\in S$$ (29)

$$T_{i}=t_{i}^{\prime }+T_{s}$$ (30)

$$t_{j}^{\prime }=\left\{ \begin{array}{c} T_{i}+\frac{d_{ij}}{v_{t}},i\in H,j\in H,x_{ij}^{k}=\text{1},k\in K\\ T_{i}+\frac{d_{ij}^{\prime }}{v_{d}},i\in (M\cup S),j\in (M\cup S) \end{array}\right.\;$$ (31)

$$T_{v}=\text{max}\left(\forall \left[\left(T_{i}+\frac{d_{is}^{\prime }}{v_{d}}\right)x_{js}^{k}y_{js}\right]|i\in N_{s},j\in G,k\in K\right)$$ (32)

$$x_{ij}^{k}=\left\{\text{0},\text{1}\right\},i\in H,j\in H,k\in K$$ (33)

$${\text{y}}_{\text{ij}}=\left\{\text{0},\text{1}\right\},i\in H,j\in H$$ (34)

$$z_{s}^{k}=\{\text{0},\text{1}\},k\in K,\text{s}\in \text{S}$$ (35)

Equation (17) represents the minimization of delivery costs; Equation (18) represents the minimization of carbon emissions; Equation (19) represents the maximization of average customer satisfaction; Equation (20) represents that each customer point can only be served once by either a vehicle or a drone, with no repeated deliveries allowed; Equation (21) ensures that the sum of the number of customer points assigned to all cluster centers equals the total number of customer points; Equation (22) ensures that each customer point belongs to only one cluster center; Equation (23) represents that the total customer demand at all vehicle stop points reached by vehicles must not exceed the maximum load capacity of the vehicles; Equation (24) represents that the total demand weight of all customers delivered by drones taking off from vehicle stop points must not exceed the maximum load capacity of the drones; Equation (25) represents that the total driving distance of vehicles from the distribution center to their return to the distribution center must not exceed the maximum endurance distance of the vehicles; Equation (26) represents that the total flight distance of drones from their departure from vehicle stop points, through customer deliveries, to their return to vehicle stop points must not exceed the maximum flight endurance distance of the drones; Equation (27) represents that vehicles depart from the distribution center and finally return to the distribution center; Equation (28) represents that drones depart from vehicle stop points and finally return to vehicle stop points; Equation (29) represents that each vehicle stop point is allowed to be visited only once. The first part of Equation (30) represents that the time when a drone leaves a customer point is equal to the sum of the time it arrives at the customer point and the service time at that customer point; the second part represents that the time when a vehicle leaves a customer point is equal to the sum of the time it arrives at the customer point and the service time at that customer point; The first part of Equation (31) represents that when a vehicle travels from node to node, the arrival time at node is equal to the sum of the departure time from node and the travel time from node to node; the second part represents that when a drone flies from node to node, the arrival time at node is equal to the sum of the departure time from node and the flight time from node to node; Equation (32) represents that the departure time of a vehicle from a stop point is the latest time when all on-board drones of that vehicle at that node return to the vehicle stop point; Equation (33) represents 1 if a vehicle travels from node to node, otherwise 0; Equation (34) represents 1 if a drone flies from node to node, otherwise 0; Equation (35) represents 1 if stop point s is delivered by vehicle k, otherwise 0..

## 3. Algorithm construction

Based on the characteristics of the problem and model solved in this paper, a multi-stage heuristic algorithm is designed to solve the vehicle-drone collaborative delivery path. The principles of the algorithm at each stage are as follows:

In the first stage, according to the coordinates of customer points, an improved K-means algorithm is used to divide all customer points into n groups. Each group has a cluster center, and each cluster center has a clustering radius, which is the maximum flight radius of the drone. The purpose is to ensure that each drone can depart from the cluster center, deliver to at least one customer point, and then return to the cluster center. The cluster center is the location where the vehicle stops, so the obtained cluster center is called a vehicle stop point.

In the second stage, based on the n cluster centers obtained in the first stage, each cluster center is analyzed in turn. Since this paper considers the path planning problem of vehicle-assisted drone delivery, drones may encounter no-fly zones and thus be unable to deliver. Therefore, the customer points of each cluster center can be divided into two categories: the first category $\varphi_{D}^{i}$ is customer points in open areas (these can be delivered by drones), and the second category $\varphi_{\text{V}}^{i}$ is customer points in no-fly zones (these cannot be delivered by drones). In this stage, path planning is performed for the first category of customer points. The specific planning method uses a genetic algorithm to ensure that each customer point in the first category is delivered exactly once. After completing the planning for the first category of customer points at each stop point in turn, the drone delivery plan for each stop point is obtained, as shown in Equation (36).


$$\gamma^{\text{i}}=\gamma_{\text{1}}^{\text{i}},\gamma_{\text{2}}^{\text{i}},\ldots ,\gamma_{\text{k}}^{\text{i}}|\gamma_{\text{e}}^{\text{i}}\subseteq \varphi_{\text{D}}^{\text{i}},\gamma_{\text{e}}^{\text{i}}\cap \gamma_{\text{g}}^{\text{i}}=\emptyset$$ (36)

In the third stage, the vehicle delivery path is planned for the first time. The planning objects are the distribution center and all cluster centers (i.e., vehicle stop points). Here, each vehicle stop point is regarded as a customer point, and each vehicle stop point has a demand (the sum of all customer demands at that stop point). The requirement is that vehicles depart from the distribution center, meet the maximum load and maximum endurance requirements of the vehicles, deliver to all vehicle stop points in sequence, and then return to the distribution center. Since the starting and ending points are fixed, and the number of stop points to be delivered is related to the delivery order of the stop points, the demand of the stop points, and the maximum endurance of the delivery vehicles, a variable neighborhood search algorithm is used in this stage. Finally, the initial vehicle delivery plan is obtained, as shown in Equation (37).


$$\text{V}{\text{P}}^{\prime }=\text{V}{\text{P}}_{\text{1}}^{\prime },\text{V}{\text{P}}_{\text{2}}^{\prime },...,\text{V}{\text{P}}_{\text{s}}^{\prime }|\text{V}{\text{P}}_{\text{s}}^{\prime }=[\text{center},...,{\text{A}}_{\text{i}},...,\text{center}]$$ (37)

In the fourth stage, the vehicle delivery path obtained in the third stage is re-planned. Specifically, the second category of customer points (those that cannot be delivered by drones) at each cluster center are arranged to be delivered by vehicles. After a delivery vehicle arrives at a vehicle stop point, drones first deliver to the first category of customer points. After all drones have completed their deliveries and returned to the vehicle, the vehicle then delivers to the second category of customer points in no-fly zones in sequence, and proceeds to the next stop point after completion. Therefore, the path planning problem in this stage is essentially a variant of the Traveling Salesman Problem (TSP) with fixed start and end points. Thus, an ant colony algorithm is used in this stage to obtain the vehicle delivery plan for each stop point, as shown in Equation (38).


$$\zeta^{\text{i}}=\zeta_{\text{1}}^{\text{i}},\zeta_{\text{2}}^{\text{i}},\ldots ,\zeta_{\text{k}}^{\text{i}}|\zeta_{\text{e}}^{\text{i}}\subseteq \varphi_{\text{V}}^{\text{i}},\zeta_{\text{e}}^{\text{i}}\cap \zeta_{\text{g}}^{\text{i}}=\emptyset$$ (38)

In summary of the algorithms in each stage, the final vehicle-assisted drone delivery path plan is obtained, as shown in Equation (39).


$$\text{VP}=\text{V}{\text{P}}_{\text{1}},\text{V}{\text{P}}_{\text{2}},...,\text{V}{\text{P}}_{\text{s}}|\text{V}{\text{P}}_{\text{s}}=[\text{center},...,{\text{A}}_{\text{i}},\gamma^{\text{i}},\zeta^{\text{i}},{\text{A}}_{\text{i}+\text{1}},...,\text{center}]$$ (39)

### 3.1 Phase 1-determining vehicle stop points using improved clustering algorithm

The traditional K-means algorithm aims to assign customer points to the nearest cluster center, minimizing the sum of squared distances from points within each cluster to their respective cluster center. However, the traditional K-means algorithm does not consider distance constraints, i.e., it does not limit the maximum distance from customer points to the cluster center. In the model of this paper, drones have a maximum endurance range. Therefore, to ensure that each drone can deliver to at least one customer and return to the cluster center, it is necessary to restrict the maximum distance from customer points to the cluster center; otherwise, there may be a customer point in a non-no-fly zone that cannot be delivered by either vehicle or drone.To address this limitation of the traditional K-means algorithm, this paper proposes the following improvements: After obtaining the clustered customer data using the traditional K-means algorithm, check whether the Manhattan distance from all customer points to their assigned cluster center is less than the drone’s delivery radius. If all points satisfy this condition, clustering is completed. Otherwise, increase the number of cluster centers, re-perform clustering, and repeat the process until the condition is met, thereby completing the clustering of all customer points. The pseudocode of the improved K-means (IKM) algorithm is shown in Table 2 below:

**Table 2:** IKM Pseudo – code.

Input:	The set of customer points N={1,2,3,…,n}, the number of cluster centers k = 1, the clustering radius r
Output:	The clustering assignment result $A=\{A_{\text{1}},A_{\text{2}},A_{\text{3}},\ldots ,A_{k}\}$, where $A_{i}\subseteq N$
1	Initialization: Randomly select k points as the initial cluster centers:$C=\left\{c_{\text{1}},c_{\text{2}},c_{\text{3}},\ldots ,c_{k}\right\}\subseteq \text{N}$
2	Assignment stage: For each point n∈N:
2.1	(1) Calculate the distance $d\left(n,c_{j}\right)$ from n to each cluster center $c_{j}\in C$
2.2	(2) If there exists $c_{j}$such that $d\left(n,c_{j}\right)\leq r$, then assign n to the nearest $c_{j}$: $j^{*}=\text{arg}\;min\;d\left(n,c_{j}\right)subject\;to\;d(n,c_{j}leqr$ Add p to $A_{j^{*}};$
2.3	(3) Otherwise, mark n as unassigned.
3	Check unassigned points: If there are unassigned points, set k=k+1, and return to step 2 for re – assignment.
4	Update cluster centers:
4.1	For each cluster center $c_{i}$: Calculate the average distance of the points assigned to $c_{i}$: $c_{i}^{\prime }=\frac{\text{1}}{|A_{i}|}\sum_{n\in A_{i}}n$
4.2	Update $c_{i}$ *to* $c_{i}^{\prime }$
5	Convergence check: If the positions of all cluster centers no longer change, that is: $\forall i,d\left(c_{i},c_{i}^{\prime }\right)<\varepsilon$ where *ϵ* is a small threshold, then stop the iteration.
6	Output the clustering assignment result *A*

### 3.2 Phase 2 – Solving drone delivery routes

In the first stage, this paper first uses the improved K-means algorithm to obtain the cluster centers, i.e., vehicle stop points. Meanwhile, the customer points belonging to each vehicle stop point are also identified. The customer points under each vehicle stop point can be divided into two categories: the first category includes customer points in open areas (these can be delivered by drones), and the second category includes customer points in no-fly zones (these are delivered by vehicles). In the algorithm of the second stage, route planning for drone delivery is conducted for each cluster center (vehicle stop point) and its associated first-category customer points.

**Fig 7:**
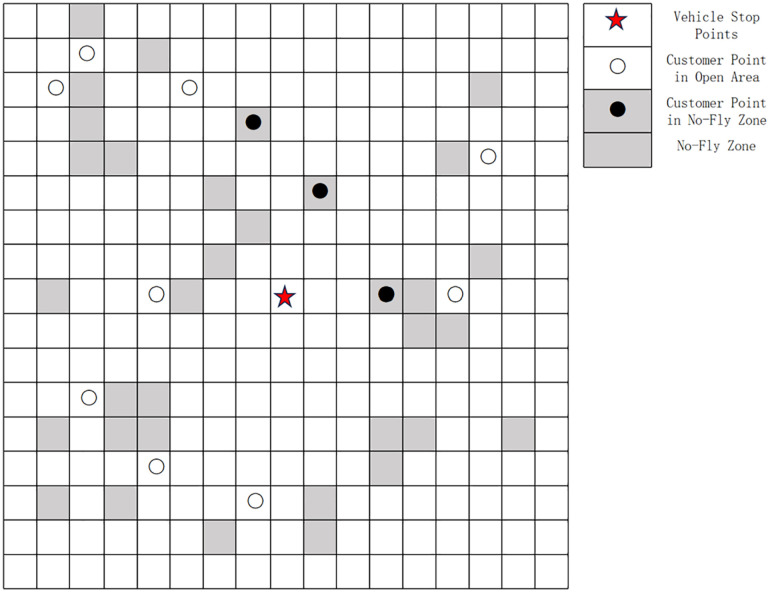
Schematic diagram of elements in the delivery area.

First, let all cluster centers (vehicle stop points) be denoted as $A=\{A_{\text{1}},A_{\text{2}},A_{\text{3}},\ldots ,A_{k}\}$, and all customer points under a cluster center be denoted as $m=\left\{m_{\text{1}},m_{\text{2}},\ldots ,m_{i},m_{i+\text{1}},\ldots ,m_{n}\right\},m_{n}\in N$. Among them, $m_{\text{1}},m_{\text{2}},\ldots ,m_{i}$ represents customer points in open areas, and $m_{i+\text{1}},\ldots ,m_{n}$ represents customer points in no-fly zones. The composition of elements within the delivery range of a cluster center is shown in Fig 7 below.

**Fig 8:**
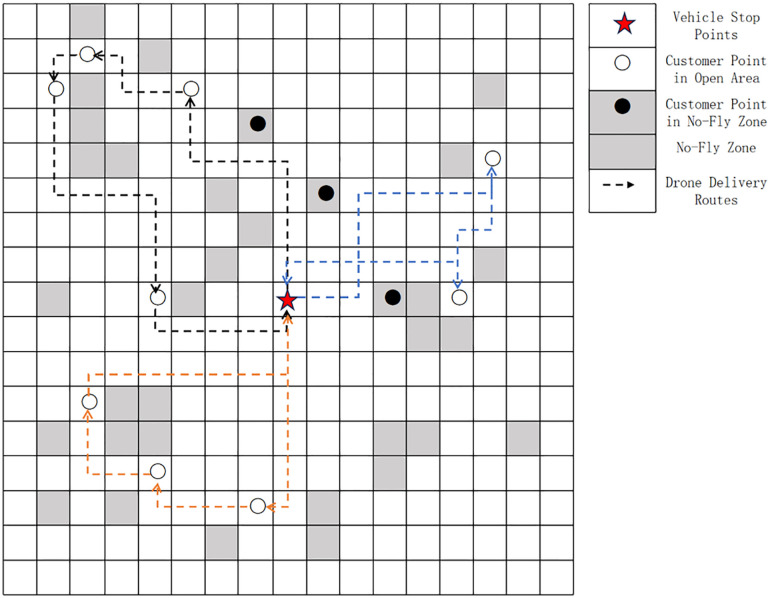
Manhattan delivery route.

As shown in Fig 7 above, for the convenience of calculation and planning, the delivery area in this paper is divided using a grid method, which facilitates marking open areas and no-fly zones. Since drones cannot deliver to customer points in no-fly zones, a detour strategy is adopted when a drone’s delivery route passes through a no-fly zone. However, regardless of the specific detour route, the flight distance is calculated using the Manhattan distance. This is illustrated in Fig 8 below:

As shown in the Fig 8, the genetic algorithm is used to plan the drone delivery scheme for cluster centers, and the pseudocode of the algorithm is presented in Table 3 below.

**Table 3:** Pseudo-code for the UAV matching rules in the third stage.

input	Maximum flight distance of the drone $L_{d}$, Maximum load capacity of the drone $W_{d}$, customer set m,
output	$Drone\_delivery\_set=[dde_{\text{1}},dde_{\text{2}},\ldots ,dde_{n}]$
1	k=1; Drone_delivery_set=[]
2	*While* k>0
3	$Cumsum\_weig\text{h}t=cumsum({[q}_{j}|j\in m])$
4	$Cumsum\_lengt\text{h}=cumsum(\left[d\left(A_{i},m(\text{1}:\text{1}),A_{i}\right),d\left(A_{i},m(\text{1},\text{2}),A_{i}\right),\ldots ,d(A_{i},m(\text{1}:n),A_{i})\right]$
5	$k=find(\text{maxf}(j)|Cumsum\_weig\text{h}t(jleqW_{d},Cumsum\_lengt\text{h}(j)\leq L_{d})$
6	*if* m=∅ *or* k=∅
7	k=k−1;
8	end
9	Drone_delivery_set[end+1]=m[1:k]*, delete* m[1:k]
10	end

According to Model Hypothesis 4, the vehicle carries multiple UAVs. However, due to the limited endurance and payload of UAVs, multiple UAVs will be used to perform the delivery tasks at the current stop. Therefore, it is first necessary to “match” the customers at this stop with the UAVs to ensure that all customers at this stop are delivered by UAVs exactly once. The matching rules are as shown in the above table. Among them, steps 3 and 4 calculate the cumulative demand and cumulative flight distance of the customer set of the initial delivery route, and step 5 finds the customer points that meet the requirements simultaneously. The specific explanations for these steps are as follows: Keep the order of the customer point set {*m*} unchanged. Starting from the first customer $m_{\text{1}}$in the set {*m*}, select one more customer point each time in order. Calculate the flight distance and payload required for the UAV to take off from the vehicle stop, deliver these customers, and then return to the stop. If it meets the rated payload and endurance of the UAV, add the customer point $m_{i}\;\;$to the delivery task of this UAV. Otherwise, end the matching of this UAV, delete the customers that have completed the matching, and continue the matching work of the next UAV among the remaining customer points until the matching of all customers in {*m*} is completed.

**Fig 9:**
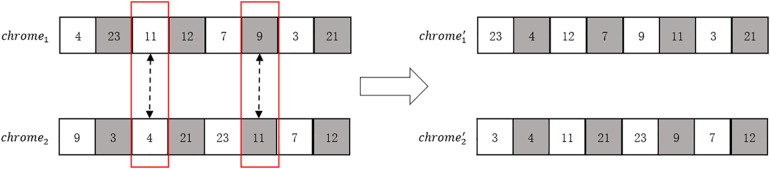
Schematic diagram of chromosome crossover.

**Fig 10:**
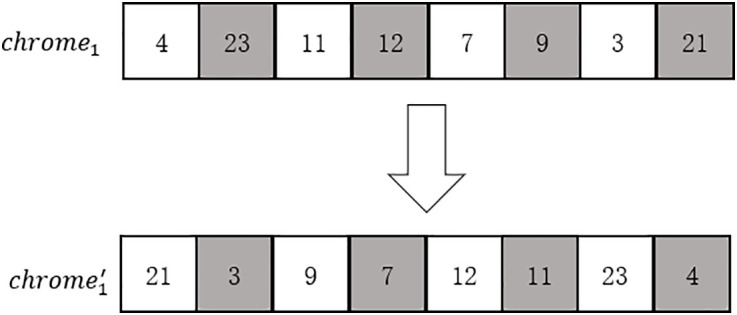
Schematic diagram of chromosome mutation.

The above – mentioned operation method is the matching principle of the UAV delivery scheme designed in this paper when the vehicle is at a stop. However, according to the above – mentioned matching method, only one UAV delivery scheme can be matched for one chromosome (here referring to the customer point set {*m*} at this stop). In this paper, the optimization goals are to minimize the delivery cost and maximize the customer satisfaction, and both of these two optimization goals are related to the flight distance of UAVs. Therefore, next, with the goal of minimizing the flight distance of the UAV delivery route, the genetic algorithm is used for the “chromosome” at this stop to find the optimal UAV delivery route. The specific steps of using the genetic algorithm for solving are as follows:

(1) Initial chromosome encoding: Use the customer numbers of the customer point set {*m*} at the stop as the initial chromosome encoding.
(2) Initialize the population: Randomly generate *n* chromosomes by shuffling the order of the initial chromosome as the initial population.
(3) Crossover: Determine the chromosome crossover probability *cr*, and perform crossover operations on the *n* populations in sequence. The crossover rule is to cross with the adjacent population, using the two – point crossover method. The crossover rule is shown in Fig 9.

(4) Mutation: Determine the chromosome mutation probability *mr*, and judge whether each population mutates in sequence. If so, reverse the order of the population, as shown in Fig 10.

(5) Selection: Combine the parent population and the offspring population after crossover and mutation, and use the roulette wheel selection strategy to re – select *n* populations from these 2*n* populations as the parent population for the next iteration.
(6) Check whether the number of iterations meets the preset maximum number limit. If it does, output the result; otherwise, go to step 3.

### 3.3 Phase 3 – Solving initial vehicle delivery routes

In the third stage of the algorithm, the path planning problem considered in this paper can be described as follows: a delivery scenario consisting of one distribution center and multiple vehicle stop points, where each delivery vehicle is required to depart from the distribution center, reach the vehicle stop points in sequence, and finally return to the distribution center. Here, vehicle stop points can be regarded as “customer points,” and the demand quantity of each vehicle stop point varies because the number of customers included in each vehicle stop point and the demand quantity of each customer are different. However, what remains consistent is that each delivery vehicle has the same load capacity and endurance mileage. Therefore, the objective of the third stage is to determine a set of vehicle delivery plans (with at least one vehicle delivery route in the plan). Each vehicle delivery route consists of the distribution center and vehicle stop points. Starting from the distribution center and finally returning to it, each vehicle must not exceed its maximum load during delivery, on the basis that all vehicle stop points are served by exactly one vehicle, and the total driving distance of each vehicle is minimized.

At this stage, with the optimization objectives of minimizing distribution cost, minimizing carbon emissions, and maximizing customer satisfaction, the specific steps for solving using the genetic algorithm are as follows:

(1) Initial chromosome encoding: The stop numbers are used as the initial chromosome encoding.
(2) Initial population initialization: Randomly generate n chromosomes with the order of initial chromosomes shuffled as the initial population.
(3) Crossover: Determine the chromosome crossover probability, and perform crossover on each population in sequence.
(4) Mutation: Determine the chromosome mutation probability, and judge whether each population mutates in sequence. If yes, reverse the order of the population.
(5) Selection: Calculate the fitness of the offspring population (including distribution cost, carbon emissions, and satisfaction), merge the parent population and the offspring population into a Pareto optimal solution set, and select the top n populations from these 2n Pareto solutions as the parent population for the next iteration.
(6) Check whether the number of iterations meets the preset maximum number of iterations limit. If satisfied, output the first solution on the Pareto frontier as the optimal solution; otherwise, return to Step 3.

### 3.4 Phase 4 – Solving the complete vehicle-assisted drone delivery route

The third stage focuses on researching the vehicle delivery routes between the distribution center and multiple vehicle stop points. Its purpose is to conduct the initial planning of vehicle delivery routes, facilitating subsequent adjustments to the routes. Specifically, it involves planning the vehicle delivery route scheme for the second category of customer points (customers in no-fly zones, who can only be delivered by vehicles) at the cluster centers from the first stage.

**Fig 11:**
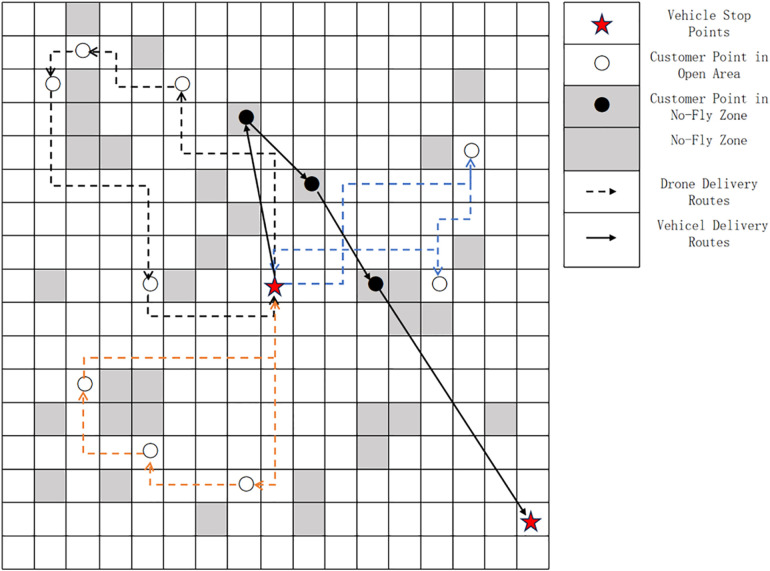
Delivery route diagram of Stage 4.

First, according to the assumptions in this paper, after a vehicle arrives at a vehicle stop point, it first uses drones to deliver to customers in open areas. Only when the drones complete delivery to the last customer in the open area of that stop point and return to the vehicle will the vehicle start delivering to the customer points in the no-fly zone of the stop point. Finally, the vehicle proceeds to the next vehicle stop point, as shown in Fig 11 below.

However, when planning the vehicle delivery scheme for customer points in the no-fly zone of a vehicle stop point, there are three scenarios as follows:

When the number of customer points in the no-fly zone of the vehicle stop point is 0, the optimal feasible solution for Stage 4 is jointly formed by the current vehicle stop point $A_{i}$ and the next vehicle stop point $A_{i+\text{1}}$, which is ${\zeta_{\text{1}}=[A}_{i},A_{i+\text{1}}]$;

When the number of customer points in the no-fly zone of the vehicle stop point is 1, the optimal feasible solution for Stage 4 is jointly formed by the current vehicle stop point $A_{i}$ and the next vehicle stop point $A_{i+\text{1}}$, which is ${\zeta_{\text{2}}=[A}_{i},\{{\text{m}}_{\text{i}+\text{1}}\},A_{i+\text{1}}]$;

When the number of customer points in the no-fly zone of the vehicle stop point is greater than 1, the feasible solution $\zeta_{\text{3}}={[A}_{i},\{m_{i+\text{1}},\ldots ,m_{n}\},A_{i+\text{1}}]$ is jointly formed by the current vehicle stop point $A_{i}$, the set of customers in the no-fly zone of the current vehicle stop point $m_{i+\text{1}},\ldots ,m_{n}$, and the next vehicle stop point $A_{i+\text{1}}$.

For the first two cases, the optimal feasible solutions are $\zeta ={[A}_{i},A_{i+\text{1}}]$ and $\zeta ={[A}_{i},\{{\text{m}}_{\text{i}+\text{1}}\},A_{i+\text{1}}]$; For the third case, this feasible solution is essentially a Traveling Salesman Problem (TSP) with fixed start and end points. In this case, it is necessary to use the genetic algorithm to solve for the optimal distribution route in. With the optimization objectives of minimizing distribution cost, minimizing carbon emissions, and maximizing customer satisfaction, the specific steps for solving the third case using the genetic algorithm are as follows:

(1) Initial chromosome encoding: Use the numbers of the second-type customer points at the stop as the initial chromosome encoding.
(2) Initial population initialization: Randomly generate n chromosomes with the order of the initial chromosomes shuffled as the initial population.
(3) Crossover: Determine the chromosome crossover probability, and perform crossover on each population in sequence.
(4) Mutation: Determine the chromosome mutation probability, and judge whether each population mutates in sequence. If yes, reverse the order of the population.
(5) Selection: Calculate the fitness of the offspring population (including distribution cost, carbon emissions, and satisfaction), merge the parent population and the offspring population into a Pareto optimal solution set, and select the top n populations from these 2n Pareto solutions as the parent population for the next iteration.
(6) Check whether the number of iterations meets the preset maximum number of iterations limit. If satisfied, output the first solution on the Pareto frontier as the optimal solution; otherwise, return to Step 3.

The above algorithm steps are for the analysis of a single stop. The output optimal feasible solutions include the following three types: $\zeta_{\text{1}}={[A}_{i},A_{i+\text{1}}]$, $\zeta_{\text{2}}={[A}_{i},\{{\text{m}}_{\text{i}+\text{1}}\},A_{i+\text{1}}]$, or $\zeta_{\text{3}}={[A}_{i},\{m_{i+\text{1}},\ldots ,m_{n}\},A_{i+\text{1}}]$. Merge the obtained optimal distribution route for the second-type customer points into the complete distribution route to obtain the final complete vehicle-assisted UAV distribution route.

## 4. Comparative analysis of numerical examples

### 4.1 Simulation analysis of small-scale instance

In the existing relevant studies, there is relatively little research on the vehicle-assisted UAV (Unmanned Aerial Vehicle) delivery route optimization problem considering no-fly zones, and it is quite difficult to obtain actual data. To verify the applicability and effectiveness of the model proposed in this paper under real-world conditions, a partial area of Harbin City, Heilongjiang Province, China was selected as the delivery area. The distribution center is Harbin International Container Central Station, with coordinates of (34.9, 40.2). Meanwhile, there are 30 customer points in different sub-areas. The desensitized coordinates, demand quantities, and time windows of these customer points are shown in Table 4 below.

**Table 4:** Basic information of customer points.

	Area	Customer Type	Actual Name	Desensitized Coordinates (x, y)	Demand Quantity	Time Window (Hour)
1	Xiangfang	Logistics Node	Yunda Yanfu Company	(8.2, 12.5)	8	9:00-17:00
2	Xiangfang	E-commerce Industrial Park	ZTO Harbin E-commerce Industrial Park	(19.7, 7.8)	15	8:00-20:00
3	Xiangfang	Large-scale Supermarket	Lesong Shopping Mall	(6.5, 23.1)	10	9:00-18:00
4	Nangang	Transportation Hub	Harbin Railway Station	(28.9, 15.4)	3	7:00-19:00
5	Nangang	Commercial Complex	Yuanda Shopping Center (Nangang Branch)	(35.2, 8.3)	12	10:00-19:00
6	Nangang	University	Harbin Institute of Technology (1st Campus)	(22.3, 29.7)	2	8:00-16:00
7	Daoli	Commercial Core Area	Central Avenue Pedestrian Street	(41.5, 12.8)	5	10:00-20:00
8	Daoli	Hospital	The First Affiliated Hospital of Harbin Medical University	(48.6, 7.2)	3	8:00-12:00
9	Daoli	Logistics Park	Mapletree Harbin Logistics Park	(38.4, 21.5)	18	8:00-18:00
10	Songbei	Commercial Complex	Mixc Shopping Mall	(55.1, 15.9)	10	10:00-19:00
11	Songbei	High-end Community	Hongxing Venice Manor	(62.8, 8.6)	2	9:00-17:00
12	Songbei	Scientific Research Institution	Harbin New District Science and Technology Innovation City	(59.3, 23.4)	3	8:00-16:00
13	Hulan	District-level Commercial Center	Hulan RT-Mart Supermarket	(8.7, 37.6)	8	9:00-18:00
14	Hulan	Logistics Node	Hulan District Post Distribution Center	(19.2, 31.8)	10	8:00-17:00
15	Hulan	Vocational College	Heilongjiang Agricultural Reclamation Vocational College	(6.3, 45.2)	2	8:00-16:00
16	Acheng	District-level Hospital	Acheng District People’s Hospital	(27.5, 38.9)	3	8:00-12:00
17	Acheng	Commercial Complex	Acheng Huining Shopping Plaza	(35.8, 32.5)	7	9:00-18:00
18	Acheng	Logistics Warehousing	Logistics Park of Acheng Economic Development Zone	(22.6, 46.7)	12	8:00-17:00
19	Shuangcheng	Agricultural Product Distribution Center	Shuangcheng Northeast Asia Agricultural Products Trading Center	(42.3, 37.1)	15	7:00-15:00
20	Shuangcheng	Logistics Hub	JD Logistics Asia No.1 Warehouse in Shuangcheng District	(49.8, 31.4)	20	8:00-18:00
21	Shuangcheng	Food Processing Enterprise	Shuangcheng Laodingfeng Food Factory	(38.6, 45.8)	6	9:00-17:00
22	Daowai	Wholesale Market	Nanji International Wholesale Market	(56.2, 38.5)	10	7:00-15:00
23	Daowai	Logistics Node	Shentong Distribution Center in Daowai District	(63.5, 32.7)	12	8:00-17:00
24	Pingfang	Industrial Logistics	Pingfang District Auto Parts Logistics Park	(59.8, 46.3)	15	8:00-16:00
25	Pingfang	Large-scale Supermarket	Yonghui Supermarket (Pingfang Wanda)	(8.5, 61.2)	8	9:00-18:00
26	Xiangfang	Community Supermarket	Zhongyanghong Xiaoyueliang Supermarket (Anbu Residential Area)	19.6, 57.8)	3	8:00-20:00
27	Nangang	Community Hospital	Dacheng Sub-district Community Health Service Center	(28.3, 65.4)	2	8:00-16:00
28	Songbei	Fresh Food Distribution Center	Songbei District Dili Fresh Food Storage Center	(36.7, 59.1)	10	7:00-15:00
29	Hulan	Town-level Supermarket	Jiale Purchase Supermarket (Kangjin Town, Hulan District)	(48.9, 63.7)	4	9:00-17:00
30	Acheng	Town-level Logistics	Post Office Branch (Pingshan Town, Acheng District)	(57.2, 58.4)	3	9:00-15:00

**Fig 12:**
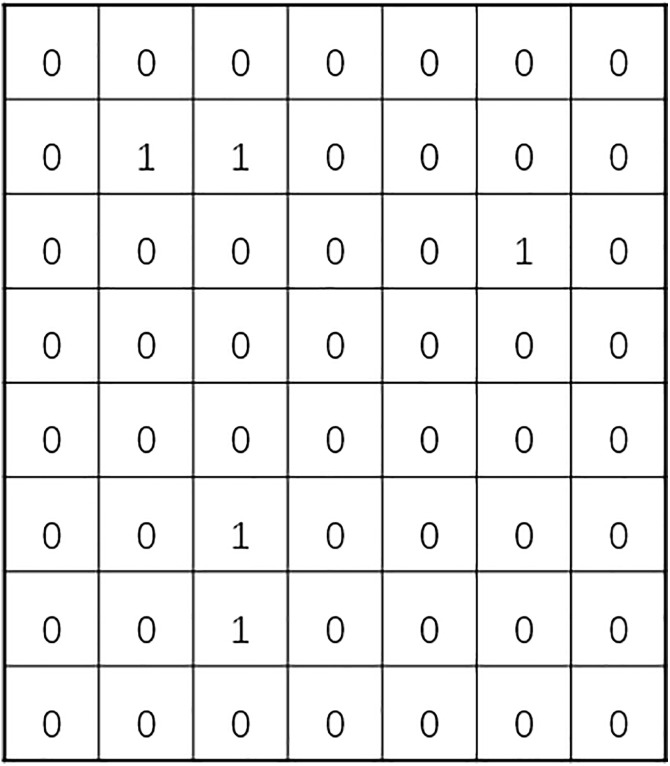
Locations of open areas and no-fly zones.

**Fig 13:**
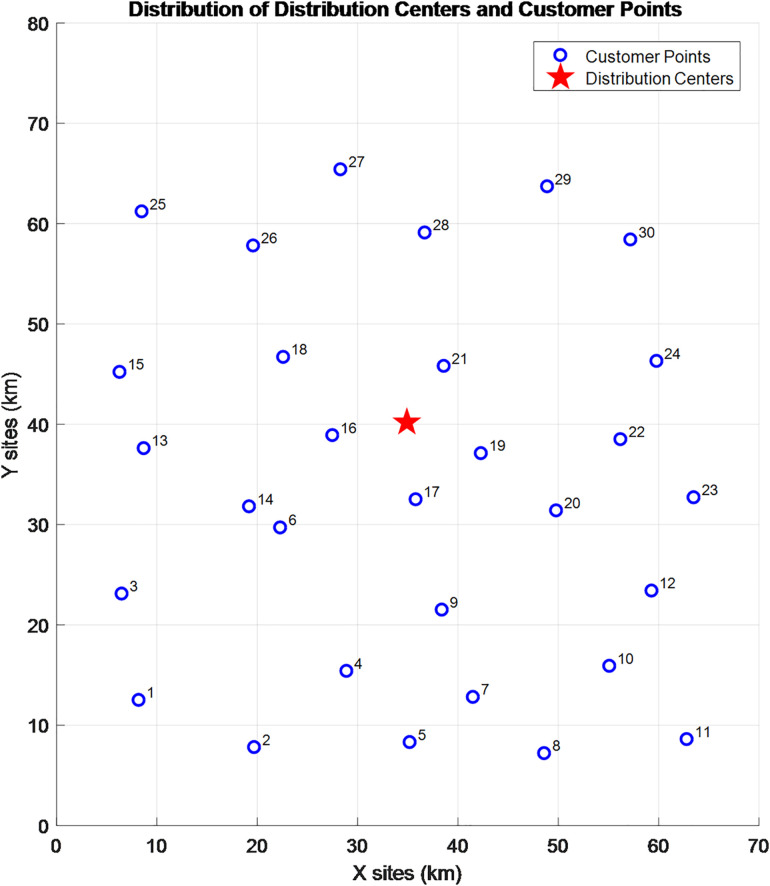
Distribution of distribution center and customer points.

Meanwhile, the delivery area was divided into 56 standard sub-areas, each with a size of 10 km × 10 km. Each sub-area is marked with 0 or 1, where 0 represents an open area and 1 represents a no-fly zone, as shown in Fig 12 below:

The specific distribution of the distribution center and customer points is shown in Figure 13 below:

A comparison between Figs 12 and 13 shows that Customer Points 4, 6, 27, and 30 are located within no-fly zones, while the remaining customer points are in open areas. Additionally, the relevant parameters in this paper are set as follows: the take-off/landing speed and time of the UAV is $\text{a}=\text{4m}/\text{s},{\text{T}}_{\text{d}}=\text{5s}$; $FC=\text{200},\theta =\text{0}.\text{15}L/km,c_{\text{0}}=\text{10},c_{\text{1}}=\text{1}.\text{5},c_{\text{2}}=\text{5},c_{\text{3}}=\text{10},W_{k}=\text{300}kg,W_{d}=\text{30}kg,V_{k}=\text{40}km/\text{h},$ $V_{d}=\text{60}km/\text{h},L_{k}=\text{200}km,L_{d}=\text{20}km,T_{s}=\frac{\text{1}}{\text{6}}\text{h}$; the departure time from the distribution center is set to 6:00. For all customers, the earliest acceptable delivery time is 2 hours earlier than the required time window, and the latest acceptable delivery time is 2 hours later than the required time window.

The algorithm was programmed using Matlab R2016b and run on a computer with a processor of 11th Gen Intel(R) Core(TM) i7-1165G7 @ 2.80GHz and 16.0 GB of memory.

#### 4.1.1 Phase 1: Analysis of clustering results.

**Fig 14:**
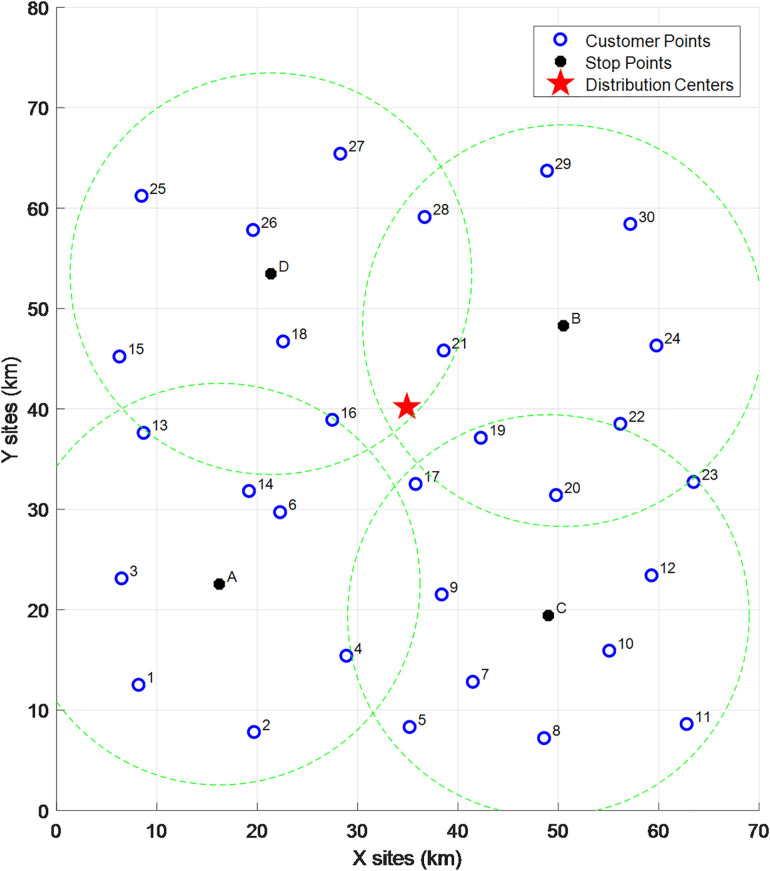
Schematic diagram of cluster centers in Phase 1.

The clustering diagram is shown in the figure below. Given the current coordinate information of customer points and the distribution center, and considering the collaborative delivery of customers by vehicle-assisted UAVs, the IKM algorithm is used to cluster 30 customer points into 4 cluster centers under the premise that the distance from all customer points to their corresponding vehicle parking points does not exceed. The location of each cluster center and its affiliated customer points are shown in Fig 14 below:

As can be seen from the Table 5, the IKM algorithm yields 4 cluster centers (A, B, C, D). The clustering coordinates (i.e., the locations of vehicle parking points) are (16.2, 22.6), (50.5, 48.3), (49, 19.4), and (21.4, 53.5) respectively. Cluster Center A covers customer points {1, 2, 3, 4, 6, 13, 14}, among which customer points {4, 6} are located in no-fly zones; Cluster Center B covers customer points {19, 21, 22, 24, 29, 30}, among which customer point {30} is located in a no-fly zone; Cluster Center C covers customer points {5, 7, 8, 9, 10, 11, 12, 17, 20, 23}; Cluster Center D covers customer points {15, 16, 18, 25, 26, 27, 28}, among which customer point {27} is located in a no-fly zone.

**Table 5:** Customer point clustering results.

Cluster Point	Clustering Coordinates	Customer Points in Open Areas	Customer Points in No-Fly Zones
A	(16.2, 22.6)	{1,2,3,13,14}	{4,6}
B	(50.5, 48.3)	{19,21,22,24, 29}	{30}
C	(49,19.4)	{5,7,8,9,10, 11,12, 17,20,23}	–
D	(21.4, 53.5)	{15,16,18, 25,26,28}	{27}

#### 4.1.2 Phase 2: UAV delivery route planning.

**Fig 15:**
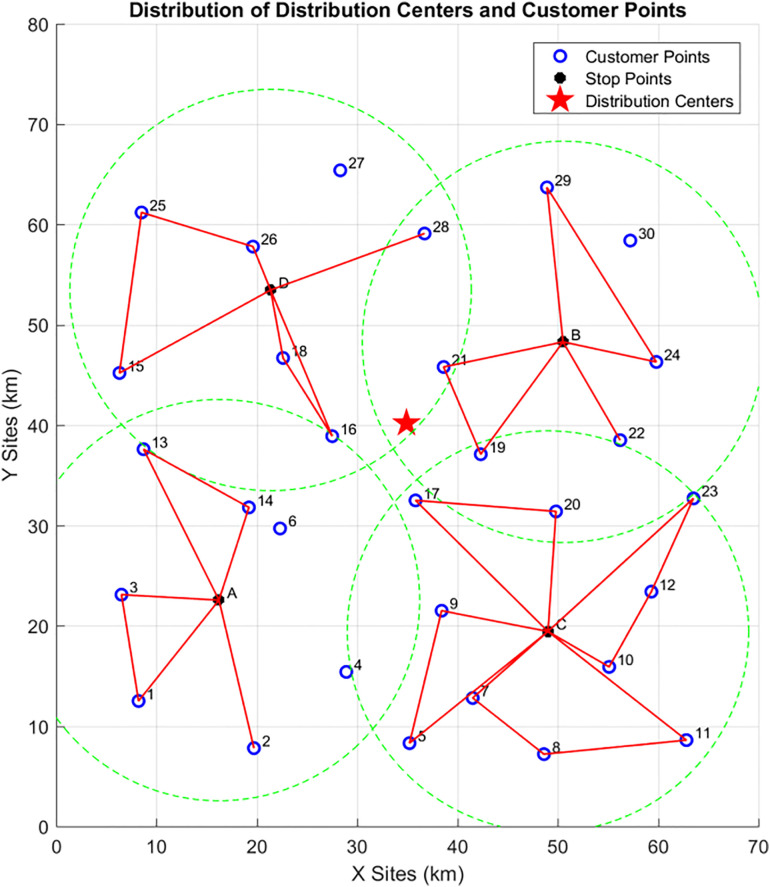
UAV delivery routes in Phase 2.

In this phase, delivery routes are planned sequentially for the customers affiliated with the four vehicle parking points (A, B, C, D). The genetic algorithm is used to solve for the UAV delivery routes, as shown in the Fig 15 (the red routes represent UAV delivery routes):At Vehicle Parking Point A: The UAV delivery routes are {A-13–14-A, A-3–1-A, A-2-A}; At Vehicle Parking Point B: The UAV delivery routes are {B-29–24-B, B-21–19-B, B-22-B}; At Vehicle Parking Point C: The UAV delivery routes are {C-23-12-10-C, C-7-8-11-C, C-5–9-C, C-17–20-C};At Vehicle Parking Point D: The UAV delivery routes are {D-15-25-26-D, D-16–18-D, D-28-D}.

#### 4.1.3 Phase 3: Initial vehicle delivery routes.

**Fig 16:**
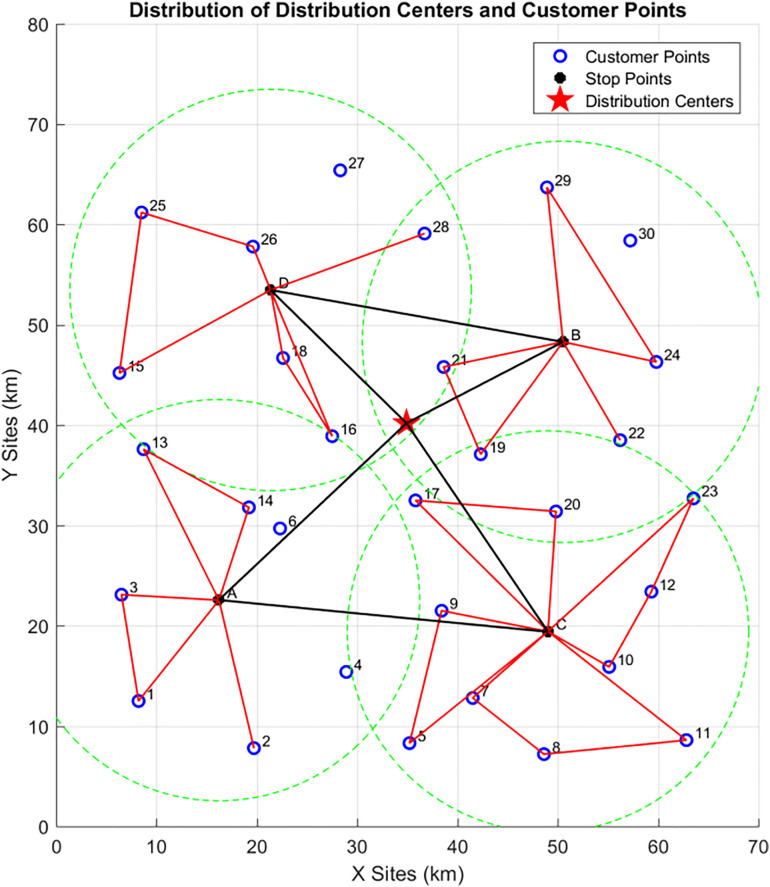
Initial vehicle delivery routes in Phase 3.

In this phase, the Variable Neighborhood Search (VNS) algorithm is used to plan the initial vehicle delivery routes. The planning objects include the distribution center and the four vehicle parking points. Two factors—maximum vehicle load capacity and endurance—are considered to comprehensively plan the number of delivery vehicles and their delivery routes. As shown in the Fig 16 below:The initial delivery route of Delivery Vehicle 1 is {0-D-B-0} (Note: “0” represents the distribution center).The initial delivery route of Delivery Vehicle 2 is {0-A-C-0}.

#### 4.1.4 Phase 4: Final vehicle delivery routes.

**Fig 17:**
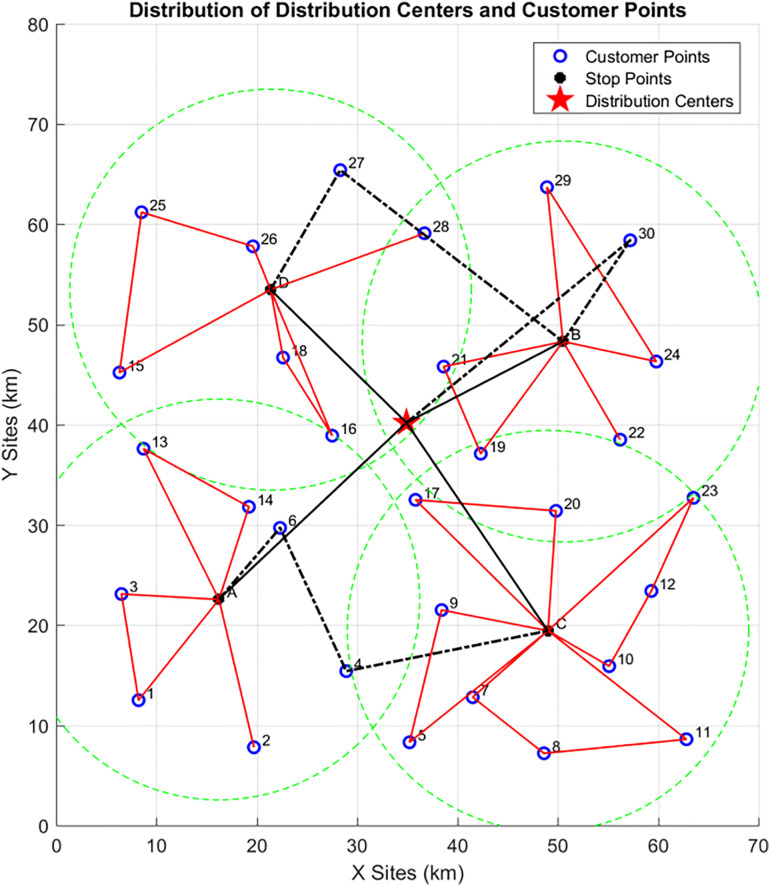
Final delivery routes of vehicle-assisted UAVs.

Based on the initial vehicle delivery routes obtained in Phase 3, it is considered that customer points in no-fly zones must be delivered by vehicles. Therefore, customers not included in the route planning of Phase 2 are assigned to vehicles for delivery. The ant colony algorithm is used to solve for the final routes of the vehicle-assisted UAV delivery system, as shown in the Fig 17 below:The final delivery route of Vehicle 1 is {0-D-27-B-30–0}.The final delivery route of Vehicle 2 is {0-A-6–4-C-0}.The optimized parts between the initial and final vehicle delivery routes are indicated by the black dashed lines in the Fig 17.

#### 4.1.5 Analysis of vehicle-assisted UAV delivery route planning results for small-scale instances.

To summarize, the algorithm-solved routes of each phase are shown in Table 6 below.

**Table 6:** Vehicle delivery routes.

Vehicl 1	Center	D	{D-15-25-26-D} {D-16–18-D} {D-28-D}	{27}	B	{B-29–24-B} {B-21–19-B} {B-22-B}	{30}	Center
Vehicl 2	Center	A	{A-13–14-A} {A-3–1-A} {A-2-A}	{6,4}	C	{C-23-12-10-C} {C-7-8-11-C} {C-5–9-C} {C-17–20-C}	Center	

Complete Delivery Route of Vehicle 1 Starting from the distribution center, Vehicle 1 first arrives at Vehicle Parking Point D. Here, 3 on-board UAVs are deployed to deliver to customer points {15, 16, 18, 25, 26, 28} in open areas. After the last UAV completes its delivery and returns to Vehicle 1, Vehicle 1 travels to customer point 27 (in a no-fly zone) for delivery. It then proceeds to Vehicle Parking Point B, where 3 on-board UAVs deliver to customer points {19, 21, 22, 24, 29} in open areas. Once the last UAV returns to Vehicle 1, Vehicle 1 travels to customer point 30 (in a no-fly zone) for delivery, and finally returns to the distribution center.

Complete Delivery Route of Vehicle 2 Starting from the distribution center, Vehicle 2 first arrives at Vehicle Parking Point A. Here, 3 on-board UAVs are deployed to deliver to customer points {1, 2, 3, 13, 14} in open areas. After the last UAV completes its delivery and returns to Vehicle 2, Vehicle 2 sequentially travels to customer points {6, 4} (both in no-fly zones) for delivery. It then proceeds to Vehicle Parking Point C, where 4 on-board UAVs deliver to customer points {5, 7, 8, 9, 10, 11, 12, 17, 20, 23} in open areas. Once the last UAV returns to Vehicle 2, Vehicle 2 returns to the distribution center.

The total final delivery cost is 2293.8, consisting of the following components:

Total vehicle delivery cost: 1582.6; Total UAV delivery cost: 580.35; Time penalty cost: 130.88; The average customer satisfaction is 0.9121. Details are shown in Table 7 below.

**Table 7:** Costs and satisfaction of vehicle-assisted UAV delivery.

Total Vehicle Travel Distance	Vehicle Delivery Cost	Total UAV Flight Distance	UAV Delivery Cost	Time Penalty Cost	Average Satisfaction	Carbon Emissions	Total Cost
118.26	1582.6	386.9	580.35	130.88	0.9121	17.89	2293.8

### 4.2 Analysis of randomly generated no-fly zones

No-fly zones are a critical factor influencing the solution results of the vehicle-assisted UAV delivery model. In this section, 10 no-fly zones are randomly generated (with all other parameters unchanged) to compare with the solution results of the benchmark zone in Table 7 above. This comparison aims to verify the stability of the model under the condition of randomly generated no-fly zones.

The data in Table 8 above are obtained from 10 runs under the two scenarios. To verify the impact of randomly generated no-fly zones on the optimization objective of vehicle-assisted UAV delivery route planning, this paper adopts the t-test significance difference method based on the MATLAB platform to test the data in Tables 5–8. Since the population mean and variance are unknown, the *Shapiro-Wilk* normality test is first used to examine the normal distribution characteristics of each sample dataset in the table. The following hypotheses are proposed:

**Table 8:** Comparison of results under different experimental scenarios.

Run Number	Experimental Scenario with Random No-Fly Zones				Benchmark Scenario			
	F	Ω	P	Cput(s)	F’	Ω’	P’	Cput’(s)
1	2264.73	17.61	0.9123	78	2293.81	17.89	0.9121	78
2	2254.32	17.54	0.9148	76	2254.33	17.34	0.9134	79
3	2210.54	16.93	0.9147	75	2302.19	18.23	0.9124	72
4	2256.72	17.18	0.9158	71	2210.92	17.23	0.9183	85
5	2229.01	17.17	0.9148	79	2248.94	17.19	0.9172	72
6	2235.71	17.29	0.9122	82	2275.98	17.54	0.9128	76
7	2281.13	17.54	0.9145	73	2189.04	17.03	0.9147	69
8	2231.56	17.34	0.9171	72	2215.67	17.18	0.9128	83
9	2309.5	18.13	0.9128	71	2254.79	17.29	0.9148	78
10	2278.35	17.23	0.9124	77	2263.92	17.46	0.9133	74

$H_{\text{0}}$: The sample data follows a normal distribution.
$H_{\text{1}}$: The sample data does not follow a normal distribution.

The confidence level is set to [specify the confidence level if available]. When h=0, p≥0.05, the $H_{\text{0}}$ hypothesis is accepted; whenh=1, p<0.05, the $H_{\text{0}}$ hypothesis is rejected. As can be seen from the test results in Table 9, each group of sample data follows a normal distribution, allowing the t-test to be used for significance testing.

**Table 9:** Shapiro-Wilk test results.

	Experimental Scenario with Random No-Fly Zones				Benchmark Scenario			
Sample Size	10				10			
Target Value	F	Ω	P	Cput(s)	F’	Ω’	P’	Cput’(s)
Mean Value	2255.15	17.39	0.9141	75.4	2250.95	17.43	0.9141	76.6
Standard Deviation	29.57	0.3305	0.0017	3.6878	36.47	0.3669	0.0021	5.0376
h	0	0	0	0	0	0	0	0
p	0.5000	0.5000	0.3720	0.5000	0.4843	0.2706	0.0874	0.5000

After verifying that the sample data conforms to the characteristics of a normal distribution, the t-test is conducted on the two groups of sample data. First, the following hypotheses are proposed for each group of data:

$H_{\text{0}}$: The algorithm in this paper has no impact on the solution under different problem instances.
$H_{\text{1}}$: The algorithm in this paper has an impact on the solution under different problem instances.

The confidence level is set to 0.05. When h=1 and p<0.05, it indicates that the null hypothesis $H_{\text{0}}$ is not valid; otherwise, the $H_{\text{0}}$ hypothesis is accepted. By conducting the t-test on six pairs of sample data, the test results are shown in Table 10 below.

**Table 10:** t-Test analysis results.

	(F,F’)	(Ω,Ω’)	(P,P’)	(Cput,Cput’)
h	0	0	0	0
p	0.7807	0.7910	0.9628	0.5516

As can be seen from Table 10, the t-test is used to analyze the stability of the vehicle-assisted UAV delivery route planning model designed in this paper.Under the condition of randomly generating no-fly zones, the solution stability of the algorithm proposed in this paper is not affected, and the H₀ hypothesis holds.

### 4.3 Comparison of delivery routes under different scenarios

**Fig 18:**
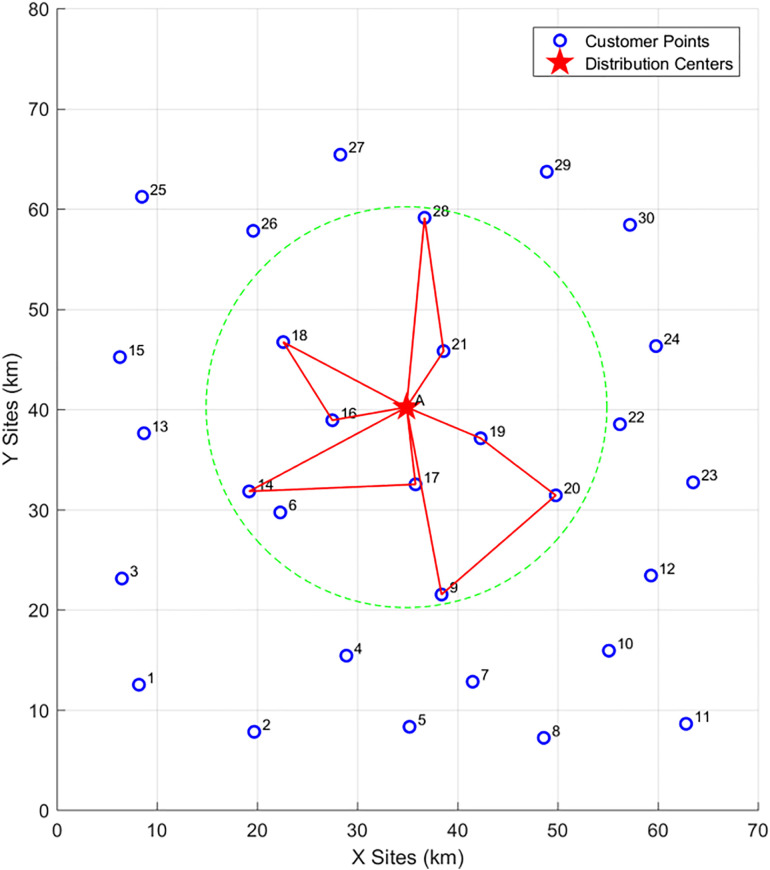
UAV-only delivery routes.

To demonstrate the differences in delivery routes and objective results under different scenarios, this paper compares three scenarios: traditional UAV-only delivery (Scenario 1), vehicle-only delivery (Scenario 2), and the proposed vehicle-assisted UAV delivery (Scenario 3). All parameters remain unchanged, and the algorithm is used to solve for the routes in Scenario 1 and Scenario 2. The route for UAV-only delivery (Scenario 1) is shown in the Fig 18 below:

In Fig 18 above, since no vehicles are involved in delivery, UAVs depart from the distribution center (denoted as “center”), deliver to customer points within their service range, and then return to the center. However, due to the limited maximum flight distance of UAVs, they can only deliver to customer points within their maximum endurance radius. As shown in the figure, a total of 4 UAVs are deployed, with the following delivery routes:{center, 21, 28, center}、{center, 18, 16, center}、{center, 14, 17, center}、{center, 9, 20, 19, center}. Customer Point 6 is within the delivery range but located in a no-fly zone, so no UAV is assigned to deliver to it.

**Fig 19:**
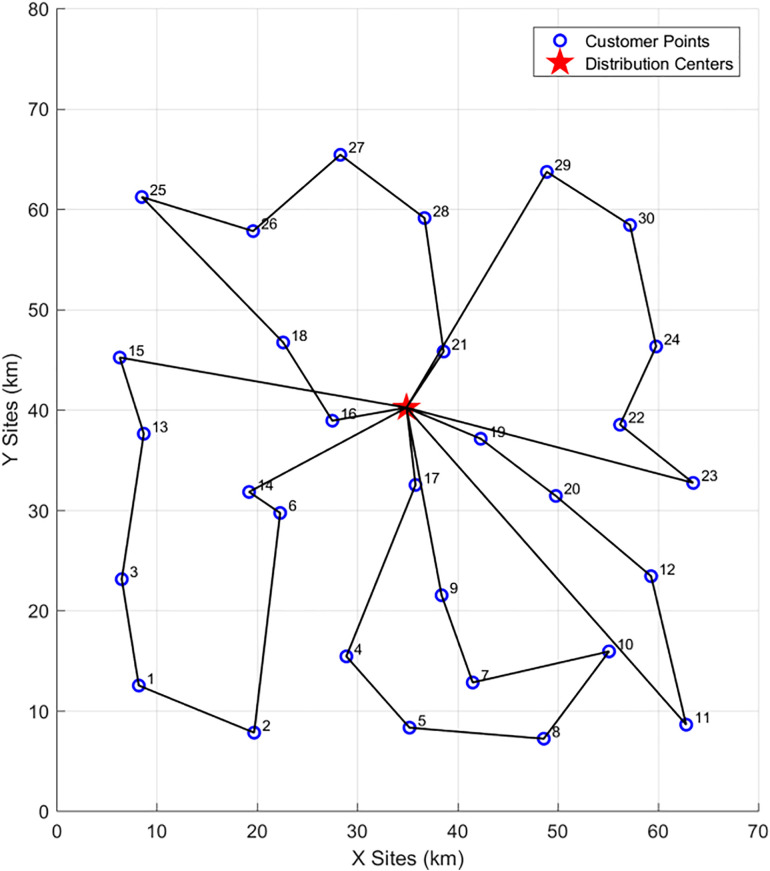
Vehicle-only delivery routes.

Meanwhile, the vehicle delivery route map for vehicle-only delivery (Scenario 2) is shown in Fig 19 below. For vehicle delivery, there is no restriction on delivery radius—only a limit on the maximum driving distance of vehicles—and no need to consider no-fly zones. Therefore, in the final planned vehicle delivery routes, all customer points are covered. A total of 5 vehicles are used for delivery in Scenario 2, with the following routes:{center, 17, 4, 5, 8, 10, 7, 9, center}、{center, 15, 13, 3, 1, 2, 6, 14, center}、{center, 16, 18, 25, 26, 27, 28, 21, center}、{29, 30, 24, 22, 23, center}、{center, 19, 20, 12, 11, center}.

The delivery route results of the three scenarios are summarized and compared in Table 11 below:

**Table 11:** Comparison of delivery route results under different scenarios.

	Vehicle Cost	UAV Cost	Total Vehicle Travel Distance	Total UAV Flight Distance	Carbon Emissions	Total Cost	Average Customer Satisfaction	Number of Delivery Vehicles	Number of Unserved Customers
Scenario 1	–	157.05	–	104.7	–	–	–	0	21
Scenario 2	2984.52	–	389.23	–	89.32	3219.79	0.8123	5	0
Scenario 3	1582.6	580.35	118.26	386.9	17.89	2293.8	0.9121	2	0

As can be seen from Table 11:In Scenario 1 (UAV-only delivery), due to the limitations of UAV flight distance and load capacity, multiple UAVs are required for delivery. Moreover, UAVs cannot reach customers beyond their maximum endurance radius, resulting in many customers being unserved—this is not in line with practical needs.In Scenario 2 (vehicle-only delivery), higher delivery costs and carbon emissions are incurred, and customer satisfaction is relatively low.In Scenario 3 (vehicle-assisted UAV delivery), the collaborative delivery of customers by vehicles and UAVs achieves the following outcomes (as indicated by objectives such as cost, carbon emissions, and customer satisfaction): it not only ensures that all customers are served but also reduces costs, promotes low-carbon operations, and improves customer satisfaction.

### 4.4 Simulation analysis of large-scale instances

#### 4.4.1 Analysis of simulation results.

**Fig 20:**
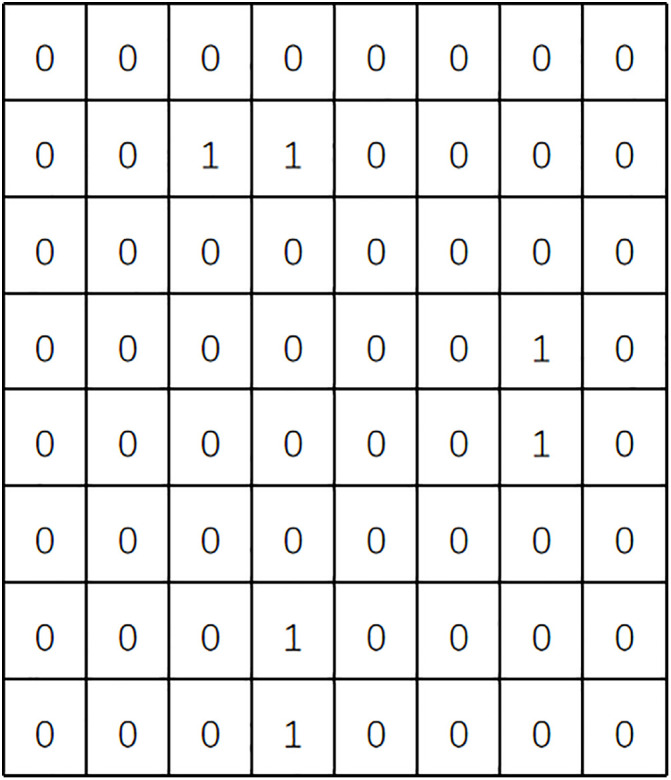
Division map of the delivery area.

In practical scenarios, enterprises typically need to serve a large number of customer points with complex characteristics. Therefore, the simulation results based on small-scale data are insufficient to demonstrate that the model and algorithm can operate stably under more complex conditions. In this subsection, the R201 dataset from the Solomon benchmark—characterized by densely clustered customer points—is selected, and the multi-stage algorithm proposed in this paper is applied to solve the route optimization problem for vehicle-assisted UAV delivery.Vehicle and UAV parameters remain unchanged. The delivery area is still divided into 10 km × 10 km rectangular sub-areas and abstracted into a 2D plane, where 0 represents an open area and 1 represents a no-fly zone. The division map of the delivery area is shown in Fig 20 below:

The algorithm program was run 10 times, and the optimal vehicle-assisted UAV delivery scheme (based on solution results) was selected.

**Fig 21:**
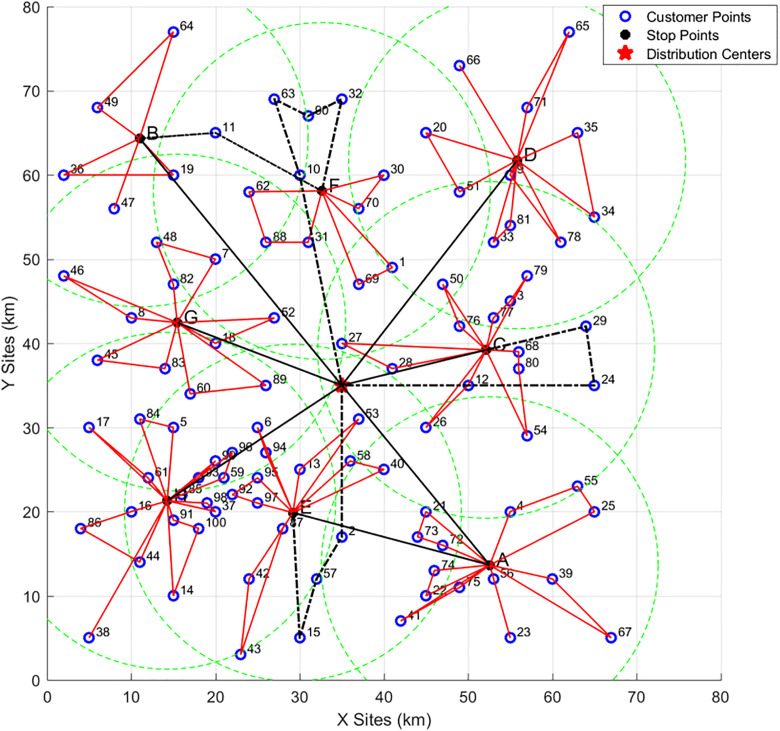
Final delivery routes for the large-scale instance.

The delivery routes are shown in the Fig 21. First, analyzing the locations of all customer points: Customer Points {2, 10, 11, 15, 24, 29, 32, 57, 63, 90} are located in no-fly zones, while the rest are in open areas. There are 8 cluster centers (i.e., vehicle parking points) with the following coordinates:A: (52.27, 13.64)、B: (11, 63.33)、C: (52.14, 39.21)、D: (55.82, 61.73)、E: (29.20, 19.87)、F: (32.64, 58.09)、G: (15.45, 42.45)、H: (14.28, 21.28). In Fig 21, Red solid lines represent UAV delivery routes.Black lines represent vehicle delivery routes, where solid black lines indicate vehicles traveling to parking points, and dashed black lines indicate vehicles traveling to customer points in no-fly zones for delivery.

The specific delivery plans for all vehicles are shown in Table 12 below. Here:Customer points in [] are delivered by UAVs. Customer points in {} are delivered by vehicles.The order from left to right in the table indicates the vehicle’s delivery sequence. For example: Vehicle 2 departs from the distribution center (denoted as “0”), first arrives at Parking Point B. UAVs are then deployed to deliver to [64, 49], [19, 36], and [47]. Next, Vehicle 2 delivers to customer point {11} (in a no-fly zone), then proceeds to Parking Point F. UAVs at F deliver to [30, 70], [1, 69], and [31, 88, 62]. After that, Vehicle 2 sequentially delivers to customer points {32, 90, 63, 10} (all in no-fly zones) and finally returns to the distribution center.

**Table 12:** Delivery plans for different vehicles.

Vehicle 1	0	A	[4,55,25],[39,67], [41,75],[21,73,72], [22,74],[23]	E	[58,40],[13,53], [87,43,42],[6,94], [95,92,97]		{15,57,2}	0
Vehicle 2	0	B	[64,49],[19,36], [47]	{11}	F	[30,70],[1,69], [31,88,62]	{32,90,63,10}	0
Vehicle 3	0	C	[12,26],[27,28],[68,80,54],[77,3,79],[50,76]				{29,24}	0
Vehicle 4	0	D	[71,65],[35,34],[81,33],[20,51],[78,9],[66]					0
Vehicle 5	0	G	[7,48,82],[46,8],[45,83],[60,89],[18,52]					0
Vehicle 6	0	H	[5,84],[61,17],[16,86,44],[91,100,14], [37,98],[85,59,96],[99,93],[38]					0

The total final delivery cost is 7276.03, consisting of the following components:Total vehicle delivery cost: 6009.67, Total UAV delivery cost: 964.8, Time penalty cost: 301.56, The average customer satisfaction is 0.9037, with details shown in Table 13 below:

**Table 13:** Costs and satisfaction of vehicle-UAV collaborative delivery.

Total Vehicle Travel Distance	Vehicle Delivery Cost	Total UAV Flight Distance	UAV Delivery Cost	Time Penalty Cost	Average Satisfaction	Carbon Emissions	Total Cost
489.67	6009.67	643.2	964.8	301.56	0.9037	157.4	7276.03

#### 4.4.2 Analysis of UAV load capacity.

In the sensitivity analysis of the large-scale instance simulation, this subsection focuses on how the UAV load capacity constraint affects the optimization results of the proposed model and algorithm. To test the impact of UAV load capacity $W_{d}$ on vehicle-UAV collaborative delivery, $W_{d}$ was set to 30, 35, 40, 45, and 50 for testing. The vehicle-assisted UAV delivery model was run 10 times, and the optimal solution was selected. The results are shown in Table 14 below:

**Table 14:** Cost comparison under different UAV load capacities.

$W_{d}$	30	35	40	45	50
Number of Parking Points	8	8	8	8	8
Number of Deployed Vehicles	6	6	6	6	6
Total UAV Flight Distance	643.2	608.1	583.4	520.8	483.7
UAV Delivery Cost	964.8	912.15	875.1	781.2	725.55
Fixed Vehicle Cost	1200	1200	1200	1200	1200
Total Vehicle Travel Distance	489.67	489.67	489.67	489.67	489.67
Vehicle Delivery Cost	6009.67	6009.67	6009.67	6009.67	6009.67
Time Penalty Cost	301.56	301.56	301.56	301.56	301.56
Carbon Emissions	157.4	157.4	157.4	157.4	157.4
Average Customer Satisfaction	0.9037	0.9018	0.8981	0.8967	0.8928
Total Cost	7276.03	6921.82	6884.77	6790.87	6735.22

As observed from Table 14, UAV load capacity directly affects total UAV flight distance and customer satisfaction. Specifically:A smaller maximum UAV load capacity means UAVs can serve fewer customers per trip, requiring more UAV deployments at vehicle parking points. For example:When $W_{d}=\text{40}$: The total UAV flight distance is 583.4 km, the UAV delivery cost is 875.1, the total cost is 6884.77, and the average customer satisfaction is 0.8981. However, A larger maximum UAV load capacity allows UAVs to carry more weight and serve more customers per trip, reducing total UAV flight distance, but average customer satisfaction decreases.

#### 4.4.3 Analysis of UAV endurance range.

The UAV endurance range $L_{d}$ is another key constraint limiting delivery operations. Keeping other parameters unchanged, $L_{d}$ was set to 30, 35, 40, 45, and 50 to study their impact on vehicle-assisted UAV delivery routes. The experimental results are shown in Table 15. As observed from Table 15, changes in maximum UAV endurance directly affect total UAV flight distance, total vehicle travel distance, total delivery cost, carbon emissions, and average customer satisfaction. This is because UAV endurance directly determines the clustering radius in the first stage of the algorithm: a larger endurance allows a larger clustering radius (resulting in fewer cluster centers) and vice versa, which further impacts the optimization results of subsequent algorithm stages. Specific observations:A smaller maximum UAV endurance leads to a shorter clustering radius and a shorter single-trip flight distance for UAVs, meaning fewer customers can be served per UAV trip and more UAV deployments are required.

**Table 15:** Cost comparison under different UAV endurance ranges.

$L_{d}$	30	35	40	45	50
Number of Parking Points	11	9	8	6	4
Number of Deployed Vehicles	9	8	6	5	4
Total UAV Flight Distance	793.9	704.3	643.2	601.2	563.1
UAV Delivery Cost	1190.85	1056.45	964.8	901.8	844.65
Fixed Vehicle Cost	1800	1600	1200	1000	800
Total Vehicle Travel Distance	613.22	531.56	489.67	412.88	365.98
Vehicle Delivery Cost	7932.2	6915.6	6009.67	5128.8	4459.8
Time Penalty Cost	356.17	330.23	301.56	278.19	247.21
Carbon Emissions	213.47	187.61	157.4	121.21	98.76
Average Customer Satisfaction	0.8823	0.8984	0.9037	0.9067	0.9132
Total Cost	9479.22	8302.28	7276.03	6308.79	5551.66

For example:When $L_{d}=\text{30}$: The number of cluster centers (vehicle parking points) is 11, the total UAV flight distance is 793.9 km, the total delivery cost is 9479.22, the time penalty cost is 356.17, and the average customer satisfaction is 0.8823. A larger maximum UAV endurance allows longer single-trip flight distances and more customers served per trip, reducing the number of cluster centers and the total uav flight distance. For example:When $L_{d}=\text{50}$: The number of cluster centers is 4, the total UAV flight distance is 563.1 km, the total delivery cost is 5551.66, and the average customer satisfaction for all customers is 0.9132.The above analysis of maximum UAV load capacity and maximum flight distance shows that both factors have a certain impact on delivery costs. This implies that when enterprises consider adopting vehicle-assisted UAV delivery, they should select UAV models based on actual operational needs.

#### 4.4.4 Analysis of vehicle load capacity.

The maximum vehicle load capacity $W_{k}$ refers to the maximum cargo weight a vehicle can transport in a single trip. A smaller $W_{k}$ means more vehicles are required to serve the same number of customers. In the context of this paper, this translates to: in the third stage of the algorithm (vehicle route planning), if the maximum vehicle load capacity is small, vehicles may be unable to continuously serve multiple parking points. Therefore, a sensitivity analysis was conducted on vehicle load capacity, and the results are shown in Table 16 below,As observed from Table 16:A smaller maximum vehicle load capacity means vehicles can serve fewer customers per trip, requiring more vehicles to be deployed.

**Table 16:** Cost comparison under different vehicle load capacities.

$W_{k}$	200	250	300	350	400
Number of Parking Points	8	8	8	8	8
Number of Deployed Vehicles	8	7	6	5	4
Total UAV Flight Distance	643.2	643.2	643.2	643.2	643.2
UAV Delivery Cost	964.8	964.8	964.8	964.8	964.8
Fixed Vehicle Cost	1600	1400	1200	1000	800
Total Vehicle Travel Distance	589.74	544.57	489.67	438.91	398.72
Vehicle Delivery Cost	7497.4	6845.7	6009.67	5389.1	4787.2
Time Penalty Cost	179.21	243.71	301.56	369.1	409.88
Carbon Emissions	215.71	181.23	157.4	131.01	112.38
Average Customer Satisfaction	0.9122	0.9087	0.9037	0.8891	0.8681
Total Cost	8641.41	7991.73	7276.03	6723	6161.88

For example:When $W_{k}=\text{200}$: The number of deployed vehicles is 8, the vehicle delivery cost is 7497.4, the total cost is 8641.41, and the average customer satisfaction is 0.9122. A larger maximum vehicle load capacity allows vehicles to travel longer distances and serve more customers per trip, reducing the number of deployed vehicles; When $W_{k}=\text{200}$, The number of deployed vehicles is 4, and the vehicle delivery cost is 4787.2. However, average customer satisfaction decreases to 0.8681.Additionally, as the maximum vehicle load capacity increases, vehicle carbon emissions gradually decrease. This is because although individual vehicle travel distances increase, the number of deployed vehicles is significantly reduced—lowering the total vehicle travel distance, reducing fuel consumption, and thus decreasing carbon emissions. The reason for the decrease in average customer satisfaction (despite increased vehicle capacity) is that the increased number of customers served per vehicle trip may delay delivery times for some customers, leading to lower satisfaction.

## 5. Conclusions and outlook

This study focuses on the optimization of vehicle-assisted UAV delivery paths under the constraints of no-fly zones and draws the following core conclusions: No-fly zones are practical constraints that must be given prioritized consideration in the route planning of vehicle-assisted UAV delivery. The planning model constructed based on these constraints enables differentiated delivery of customer points — with customer points in no-fly zones delivered by vehicles and those in open areas delivered by UAVs assisted by vehicles. This approach ultimately ensures full coverage of delivery for all customer points, significantly reduces the operational input costs of logistics enterprises, and effectively improves the overall average customer satisfaction.

The multi-stage heuristic solution algorithm designed in this study exhibits excellent performance. By decomposing the problem in stages (customer clustering, UAV path optimization, initial vehicle path planning, and final vehicle path optimization), the algorithm significantly reduces computational complexity. It not only shortens the solution time but also achieves better objective results (such as lower total cost and carbon emissions). Even when dealing with large-scale instances like the R201 dataset from Solomon, the algorithm can still efficiently obtain the optimal delivery plan.

Through the comparison of three delivery scenarios, it is found that UAV-only delivery fails to cover some customer points due to limitations in endurance, load capacity, and no-fly zones; while vehicle-only delivery can achieve full customer coverage, it incurs higher delivery costs and results in lower customer satisfaction. In contrast, the vehicle-assisted UAV collaborative delivery model can effectively balance these three aspects, achieving the dual goals of reducing delivery costs and improving customer satisfaction while ensuring delivery to all customer points.

Furthermore, the core parameters of equipment have a significant impact on delivery path planning and optimization results. For UAVs: in terms of load capacity, a larger load capacity allows more customer points to be delivered in a single trip, which may shorten the total flight distance but may lead to a slight decline in customer satisfaction; in terms of endurance, longer endurance reduces the number of customer cluster centers (vehicle parking points) and the number of vehicles required, thereby decreasing the total vehicle travel distance and carbon emissions while improving customer satisfaction. For vehicles: in terms of load capacity, a larger load capacity enables more delivery demands to be carried in a single trip, reducing the number of vehicles to be dispatched and lowering vehicle delivery costs and carbon emissions. However, the increased number of customer points delivered in a single trip may cause delays in delivery time for some customers, leading to a slight decline in customer satisfaction.

This paper has made certain progress and achievements in studying the optimization problem of the joint delivery route of vehicles and UAVs considering the constraints of no – fly zones. In the future, more in – depth research can be carried out from the following aspects:

First, this paper only considers one distribution center, but there may be multiple distribution centers in actual application scenarios. The design of the vehicle – assisted UAV delivery route planning for multiple distribution centers needs further in – depth research.

Second, this paper only considers one type of delivery vehicle and one type of UAV. In future research, scenarios of multi – type vehicle and multi – type UAV joint delivery need to be added and studied in depth.

Third, this paper assumes that the speeds of vehicles and UAVs remain constant during the entire delivery process. However, the real road network situation is complex and changeable, and the speeds of vehicles and UAVs are greatly affected by traffic conditions. The optimization problem of the joint delivery route of vehicles and UAVs with dynamic driving and flying speeds remains to be further studied.

Fourth, the designed four-stage heuristic algorithm (clustering → UAV path → initial vehicle path → final vehicle path) reduces the solution difficulty by decomposing complex problems. However, this strict sequential execution strategy may lead to the issue of “local optimality”. Future researchers are encouraged to explore whether information feedback or iterative mechanisms can be introduced between stages to investigate the possibility of improving solution quality.

Fifth, when calculating UAV paths, this paper explicitly adopts the Manhattan distance and “does not discuss how to find obstacle avoidance paths, but only focuses on the distance calculation of such paths”. Nevertheless, in complex no-fly zone environments, the length of actual detour paths may be much longer than the Manhattan distance, which will directly affect the accuracy of cost, time, and energy consumption calculations. Future scholars are expected to propose more accurate obstacle avoidance and pathfinding algorithms (such as the A* algorithm) to improve the UAV detour route model in this paper.

## Supporting information

S1 Data (XLSX)
